# Cartilage intermediate layer protein inhibits ligamentum flavum hypertrophy mediated by TGF-β1/SMAD3/SERPINE2 signaling pathway

**DOI:** 10.1007/s00018-025-06051-7

**Published:** 2026-02-09

**Authors:** Jiale Dong, Peng Li, Longxiao Wu, Saifei Meng, Guiwang Liu, Xiaoming Chen, Guiqing Wang, Chunlei Liu

**Affiliations:** 1 Department of Orthopedics, Division of Spine Surgery, The Affiliated Qingyuan Hospital (Qingyuan Peoples Hospital), Guangzhou Medical University, Guangdong, 511518 China; 2 Department of Spine Surgery, The Sixth Affiliated Hospital, Sun Yat-sen University, Guangdong, Guangzhou, P.R. China

**Keywords:** Cartilage intermediate layer protein, Ligamentum flavum hypertrophy, TGF-β1, SMAD3, Fibrosis

## Abstract

Lumbar Spinal Stenosis (LSS), a degenerative disorder, greatly impacts the elderly, often leads to discomfort, neurological problems, and a diminished quality of life. Ligamentum flavum hypertrophy (LFH), a marked influencor in LSS, is characterized by fibrosis resulting from excessive extracellular matrix deposition, largely driven by the differentiation of fibroblasts and inflammatory processes. Transforming growth factor β1 (TGF-β1) is pivotal in the LFH advancement by promoting fibrosis, highlighting its potential as a target for therapeutic strategies. Cartilage intermediate layer protein (CILP), known to regulate TGF-β1 activity in other tissues, may have potential in mitigating LFH. This research explores the function of CILP in LFH through the use of sophisticated bioinformatics, human samples, and experimental models, identifying its regulatory influence via the TGF-β1/SMAD3/SERPINE2 pathway.

**Supplementary Information:**

The online version contains supplementary material available at 10.1007/s00018-025-06051-7.

## Introduction

Lumbar Spinal Stenosis (LSS) has emerged as a prevalent spinal pathology significantly impacting the health of the elderly population, which is estimated that approximately 103 million individuals globally are afflicted [1]. This degenerative condition is attributed to factors such as ligamentum flavum hypertrophy (LFH), intervertebral disc degeneration (LDH), and degeneration of facet joints [2]. These abnormal alterations lead to a decrease in the volume of the spinal canal, which in turn applies stress on the spinal cord or nerve roots, causing neurological symptoms [3]. LSS results in significant neurological symptoms such as nerve numbness, pain, lower back discomfort, and intermittent claudication in the lower extremities, substantially impair motor function and diminish quality of life [4]. In the past 30 years, the number of people undergoing decompression and fusion surgery for lumbar spine diseases has tripled [5]. Current research predominantly identifies LFH as the principal pathological factor contributing to the development of LSS [6, 7].

The fundamental characteristic of LFH is the development of a pronounced fibrotic lesion [8]. Typically, the normal ligamentum flavum (LF) predominantly consists of elastic fibers organized in a mesh-like pattern, interspersed with a minor proportion of collagen fibers and fibroblasts [9]. This structural organization imparts the LF with significant elasticity and resilience, thereby facilitating its normal physiological function. Age-related alterations and mechanical stress in the spine can trigger the transformation of fibroblasts in the LF into myofibroblasts, subsequently may cause fibrosis [10, 11]. These fibroblasts contribute to the secretion of various elements of the extracellular matrix (ECM), initiating LFH and promoting collagen deposition [12].

Inflammation is critical to fibrosis development, and the extent of the inflammatory response is strongly correlated with the severity of fibrosis in the LF [13]. Transforming growth factor β1 (TGF-β1), recognized as a pro-fibrotic cytokine, has been implicated in fibrotic processes across various organs and tissues [14, 15]. Previous research has demonstrated that the expression level of TGF-β1 is notably upregulated in LFH patients. This contributes to the abnormal deposition of ECM, thereby exacerbating the progression of the disease [16]. However, identifying novel TGF-β1 inhibitors that do not interfere with physiological function remains critical [17].

Cartilage intermediate layer protein (CILP) within the ECM [18] is predominantly present in the middle region of articular cartilage, along with other structures such as the meniscus, tendons, ligaments, and intervertebral discs [19]. The N-terminal region of CILP directly interacts with TGF-β1, thereby inhibiting SMAD phosphorylation and its subsequent nuclear translocation, which creates conditions conducive to the suppression of the pathological activation of the TGF-β1 signaling pathway [20]. Within intervertebral disc repair, CILP facilitates the normal remodeling of aberrant ECM [21]. Regarding cardiac fibrosis, TGF-β1 encourages the synthesis of CILP, which subsequently acts to hinder the advancement of fibrosis mediated by a negative feedback loop [22]. Nevertheless, the role and efficacy of CILP in LFH, a fibrotic condition, have not been precisely elucidated, necessitating further investigation.

In this study, we employed iTRAQ and bioinformatics methodologies to identify differential expression of CILP and fibrosis markers in lesions of the LF. Subsequent investigations utilizing human specimens, in vitro cell models, and in vivo BS mouse models indicate that CILP exerts a negative regulatory effect on LFH via the TGF-β1/SMAD3/SERPINE2 signaling pathway. This investigation endeavors to furnish a reference for clinical investigations and therapeutic strategies targeting hypertrophy of the LF.

## Methods and materials

### Ethical approval

The study was initiated following the Helsinki Declaration and obtained authorization from the Institutional Ethics Review Committee at Qingyuan Hospital, which is affiliated with Guangzhou Medical University, under the reference number 2022-KS-215. In line with established protocols, tissue and blood specimens were gathered from the Division of Spine Surgery within the Department of Orthopedics at Affiliated Qingyuan Hospital, with all patients providing written informed consent. The animal study underwent evaluation and received approval from the Experimental Animal Ethics Committee of Affiliated Qingyuan Hospital, Guangzhou Medical University (LAEC-2024-026).

### Pre-operative LF thickness measurement

An axial T2-weighted magnetic resonance imaging (MRI) scan was conducted at the Lumbar (L) 4/5 facet joint level for all patients prior to surgery, with both axial and sagittal images being acquired. The Picture Archive and Communication System (PACS) serves a crucial role in the assessment of the maximum thickness of the LF in the axial plane. In this process, each LF is evaluated independently by two qualified surgeons, ensuring that the measurements are reliable and consistent. To arrive at a definitive measurement of LF thickness, the average of the two surgeons' assessments is calculated.

### Patients and human LF tissue collection

From January to June 2024, 42 LF samples were gathered from individuals who received either the Unilateral Biportal Endoscopic Technique (UBE) or Percutaneous Endoscopic Lumbar Discectomy (PELD) for surgery on the L4/5 region. The group with LFH included 21 patients who had been diagnosed with LSS and exhibited LF thickness of 3.74 mm or greater before the operation. In contrast, the non-LFH group consisted of 21 patients diagnosed with LDH and an LF thickness of < 3.74 mm. Patients with lumbar spondylolisthesis, rheumatism, or autoimmune diseases were excluded from the study. All Tissue specimens were extracted from the posterior aspect of the lumbar facet at the L4/L5 level throughout the course of surgical interventions. Samples were systematically stored for further experimental analysis. A detailed overview of the patients' clinical characteristics and the storage conditions and intended uses of the samples were listed in Table 1 and Table S1, respectively.

**Table 1:** Characteristics of patients in the two groups

Variable	non-LFH Group (*n* = 21)	LFH Group (*n* = 21)	*P*-value
Age (years)	58.71 ± 2.80	70.33 ± 2.69	< 0.05
Gender (male: female)	8:13	10:11	-
LF thickness (mm)	3.14 ± 0.11	5.72 ± 0.26	< 0.001
Lumbar segment	L4/5	L4/5	-
Comorbidities(case)			
hypertension	12	15	-
diabetes	0	0	-
Drug	Non-steroidal anti-inflammatory drugs	Non-steroidal anti-inflammatory drugs	-

### Patient blood sample collection

Following experimental ethics, qualified and trained nurses collected 5 mL venous blood samples using endotoxin-free EDTA-coated tubes prior to surgical procedures. Following collection, the collected serum tube was left upright at room temperature for half an hour. The samples underwent centrifugation at 3000 rpm for 10 minutes at the same temperature to eliminate blood cells. Once centrifugation was complete, the supernatant was gently moved into labeled cryovials and preserved at -80 °C for subsequent molecular biology studies.

### Microarray data datasets

The dataset identified as GSE113212 was sourced from the NCBI Gene Expression Omnibus (GEO, https://www.ncbi.nlm.nih.gov/geo/). It encompasses four LFH samples collected from older males with an average age of 80 years, alongside four Non-LFH samples sourced from younger males with a mean age of 20 years. The Agilent-039494 microarray platform was utilized to generate the data, and the probe annotations were transformed into standard Gene Symbols. All samples originated from human tissue specimens.

### Isobaric tags for relative and absolute quantification (iTRAQ)

Six LF samples were examined for iTRAQ, with an equal distribution of Non-LFH and LFH groups (3 samples each). The LF samples underwent individual homogenization in liquid nitrogen, followed by lysis with RIPA buffer and ultrasonic treatment while kept on ice. Following ultrasonication, the sample underwent centrifugation at 4 °C at 12,000× g for 20 minutes, followed by the careful collection of the supernatant. The quantity of proteins in the collected supernatant then was quantified using the Bicinchoninic Acid Assay (BCA). The alkylated protein solution was ultrafiltered and then reacted with trypsin overnight. The iTRAQ-8 labeling kit (4381663, Sigma Aldrich, MO, USA) was utilised for the labeling of peptide segments. High-pH fractionation was carried out using the Dionex Ultimate 3000 High-Performance Liquid Chromatography (HPLC) system, based in California, USA, which facilitated the efficient separation of sample components. This advanced system allowed for the effective separation of various components within the sample. Following this initial fractionation process, the resulting fractions underwent further separation through a Dionex UltiMate 3000 Nano Liquid Chromatography (LC) system. The combination of these sophisticated techniques ultimately enabled the execution of reverse-phase liquid chromatography tandem mass spectrometry (RPLC-MS/MS) analysis, a powerful method for detailed characterization and quantification of the separated fractions.

### scRNA-seq analysis

Following the surgical procedure, six pairs of LF tissue samples were thoroughly rinsed with PBS. Subsequently, fragments measuring approximately 1 mm³ were meticulously cut from these samples. These tissue fragments underwent a digestion process followed by centrifugation, which facilitated the extraction of cell pellets. To ascertain the concentration of the isolated cells, the BD Rhapsody™ single cell analysis system (BD Bio Sciences) was employed. In the next phase of the analysis, the cell samples were tagged with unique identifiers using the BD Human Single-Cell Multiplexing Kit, also from the same company. The BD Rhapsody method was then utilized for cell lysis, followed by RT-PCR to synthesize cDNA. This process ultimately culminated in the preparation of cDNA libraries, which are essential for subsequent analyses. To assess the quality of these constructed libraries, the Qubit DSDNA HS Assay Kit (Q32854, Invitrogen, Carlsbad, California, USA) was employed, ensuring that the libraries met the necessary standards for sequencing. The libraries were sequenced with the HiSeq X Ten Sequencer and the HiSeq X Ten Reagent Kit V2, both provided by Illumina in California. The initial data from scRNA-seq were analyzed using R software, version 4.1.2. Gene expression data analysis was carried out with the Seurat software package, created by the Satija Lab at the New York Genome Center. Additional data filtering was performed to enhance the quality of the analysis; this involved the removal of low-quality cells, the inclusion of multiple droplets, and the exclusion of empty droplets. 

### Transcriptomic, proteomic and bioinformatics analyses

We conducted quantile normalization on the GSE113212 original dataset to ensure that the data was appropriately adjusted for comparative analysis. This normalization process was crucial for accurately identifying differentially expressed genes (DEGs) between the LFH sample and the control group. The data processing, which included quantile normalization and subsequent analyses, was executed using the R package limma within the Bioconductor 4.1 framework. This robust tool allowed us to effectively compare the gene expression profiles of the LFH samples against those of the normal controls, facilitating a clearer understanding of the biological differences between the two groups. To enhance the interpretability of our findings, we utilized platform’s annotation data to convert probe identifiers into recognizable gene symbols. This step was essential for linking our results to specific genes, thereby providing a more meaningful context for our analysis. The identification of DEGs was detected using the Limma package in R, which is well-regarded for its statistical rigor in analyzing gene expression data. For differential gene expression analysis between Non-LFH and LFH groups, we applied a t-test, which allowed us to determine the statistical significance of the observed differences. Furthermore, to account for multiple testing and reduce the likelihood of false discoveries, we adjusted the p-values using the Benjamini-Hochberg method. This adjustment is critical in genomic studies, where the risk of type I errors can be elevated due to the large number of comparisons being made.

The mass spectrometer initially acquires mass spectrometry data in the RAW format, which is subsequently converted into MGF format using Proteome Discoverer 1.4 (Version 1.4.0.288, ThermoFisher, MA, USA). MGF format files, along with the protein retrieval library, are then utilized as inputs for ProteinPilot Software 4.5 (v1656, AB Sciex, MA, USA) to facilitate mass spectrometry data retrieval.

To visualize differentially expressed genes (DEGs) and proteins (DEPs), a volcano plot was created utilizing EnhancedVolcano (version 1.18.0). The thresholds for |fold change (FC)| were established at values exceeding 1.5, along with p-value cutoffs of either 0.05 or 0.01. The Venn diagram was generated using the limma (v3.58.1). A violin plot for SERPINE2 expression was created using ggplot2 (v3.5.1). A heatmap was generated using pheatmap (v1.0.12) to visualize protein expression patterns across different conditions. To conduct functional enrichment analysis, we utilized clusterProfiler (version 4.0.5) along with org.Hs.eg.db (version 3.12.0) for the enrichment of KEGG and GO terms. The outcomes of the KEGG enrichment were illustrated through circlize (version 0.4.16), which enabled the creation of chord diagrams. Meanwhile, the results for GO enrichment were presented using ggplot2 (version 3.5.1), producing informative visualizations.

### Cells culture, treatment and transfection

LF cell were extracted from the non-LFH tissues. The removed tissue was underwent triple washes with sterile PBS (Gibco, NY, USA; 10010023) to eliminate blood residues, epidural cartilage, and adipose tissue. Following this, the LF tissue was sectioned into tiny pieces and digested with collagenase I (Biofroxx, Germany; 1904MG100) for enzymatic digestion for 90 minutes at 37°C. Following the digestion process, the tissues were thoroughly rinsed using DMEM (Gibco, NY, USA; C11995500BT) to ensure removal of any residual enzymes or debris. Subsequently, these rinsed tissues were cultured in 35 mm Petri dishes, providing an appropriate environment for cell growth, with DMEM enriched with 20% fetal bovine serum (Gibco, 10099-141C), along with 100 U/mL penicillin and 100 mg/mL streptomycin ( Gibco, 15140-122) to prevent bacterial contamination. The cultures were incubated at 37°C within a humidified incubator that provided a 5% carbon dioxide atmosphere, which is essential for optimal cell proliferation. Medium replacement was performed at 72-hour intervals to maintain nutrient availability and eliminate accumulated waste. For the subsequent experiments, only the cells that were cultured from the third to sixth passage (P3-P6) were utilized, as these stages are typically associated with enhanced growth and viability.

To induce fibrosis in LF cells *in vitro*, the cells were treated with 10 ng/mL of recombinant TGF-β1 protein (HY-P7118, MCE, NJ, USA) for 24 hours. In further experiments, cells were exposed to varying concentrations of CILP protein (10, 25, 50 ng/ml, RPC382Hu01, Cloud-Clone, TX, USA) and/or SERPINE2 protein (25 μg/ml, HY-P71085, MCE) for 24 h.

The plasmids and siRNA were obtained from Hanbio Biotechnology Co., Ltd, located in Shanghai, China. Cells were plated in 6-well dishes at starting density of 4.5× 10^4^ cells for each well. Once cells achieved 60% confluence, the h-CILP overexpression vector, h-SERPINE2 overexpression vector, h-si-CILP, and h-si-SERPINE2 were introduced into the cells with the aid of Lipofectamine 3000 reagent (Invitrogen, CA, USA). Following this, experiments were carried out after a 48-hour incubation period.

### Cell Counting Kit-8 (CCK-8)

Cell proliferation was evaluated using a Cell Counting Kit-8 (CCK-8; Beyotime, C0038). LF cells were plated in 96-well plates at 5,000 cells per well. Upon reaching approximately 80% confluency, the cells were treated with increasing doses of CILP protein and cultured for 24 hours. Subsequently, 10 μL of CCK-8 solution was added to each well, followed by a 4-hour incubation at 37°C. The 450 nm absorbance readings were acquired with a spectrophotometer (Spectrostar NANO, BMG Labtech, Offenburg, Germany). Calculate the relative viability of cells by comparing the absorbance of different groups.

### Western blot (WB)

LF tissue samples were homogenized in RIPA lysis buffer (Beyotime, P0013B, Shanghai, China) followed by ultrasonication (settings: 5 minutes duration, 600 W power, 1-s pulse-on, 1.5-s pulse-off, alarm temperature: 25°C). The lysates were centrifuged at 12,000 rpm at 4°C for 8 minutes, and supernatants were collected. For LF cells, RIPA buffer was used for 1 minute lysis, followed by centrifugation at 12,000 rpm at 4°C for 8 minutes to obtain supernatants. The protein concentration was then assessed using a BCA protein assay kit (Beyotime, P0010, Shanghai, China). The proteins obtained were denatured in a metal water bath at 95°C for 30 minutes, and subsequently, they were separated according to their molecular weight through 7.5% and 10% sodium dodecyl sulfate polyacrylamide gel electrophoresis (SDS-PAGE). Subsequently, the proteins were transferred onto polyvinylidene fluoride (PVDF; Roche Applied Science, IN, USA) membranes employing wet transfer technology. Membranes were blocked with blocking solution (Epienzyme, PS108; blocking solution : TBST = 1:4) for 30 minutes at room temperature. Primary antibody incubations were conducted overnight at 4°C using the following dilutions: CILP (Abmart, PC13954S; 1:1000), TGF-β1 (Affinity, AF1027; 1:1000), COL1A2 (Affinity, DF3549; 1:1000), α-SMA (Affinity, AF1032; 1:1000), SMAD3 (Affinity, AF6362; 1:1000), p-SMAD3 (Affinity, AF3362; 1:1000), and SERPINE2 (Abcam, ab154591; 1:1000), and GAPDH (Abcam, ab9485; 1:1000). Following primary antibody incubation, membranes were probed with Goat Anti-Rabbit IgG (H+L) HRP (Affinity, S0001; 1:6000). Protein detection on the membrane was performed using the enhanced chemiluminescence kit (Biosharp, BL520A; Shanghai, China), and images were acquired using the ChemiDoc MP chemiluminescence gel imaging system (Bio-Rad, CA, USA).

### Quantitative real-time PCR (qRT-PCR)

Total RNA was isolated from both LF tissue specimens and cultured cells with TRIzol reagent (Invitrogen, 15596026). First-strand cDNA synthesis was performed using Easyscript SuperMix reverse transcription reagents (TransGen, AE301; Beijing, China). qRT-PCR analyses were conducted on an ABI PRISM 7500 platform (Applied Biosystems, USA) with SYBR Green-based Ex Taq II master mix (Takara, RR820A; Japan). Relative gene expression quantification was determined through the 2^−ΔΔCt^ method, with GAPDH serving as the endogenous reference. All primer sequences are provided in Supplementary Table S2.

### Histological assay

Tissue samples from humans or mice were preserved in a 4% paraformaldehyde solution (BL539A, Biosharp) for 48 hours. Following this, the spine samples from mice were subjected to decalcification using an EDTA solution (BL616B, Biosharp) over the course of one month. Following these preparatory steps, the specimens were embedded in paraffin blocks and sectioned into 4-μm thick slices using a microtome (RM2235, Leica, Wetzlar, Germany). After the processes of dewaxing and hydration, the tissue samples were stained using various kits from Solarbio, Beijing, China. These included the hematoxylin and eosin (H&E) staining kit (G1005, ServiceBio, Wuhan, China), the Masson trichrome staining kit (C0189S, Beyotime, Shanghai, China), or the Verhoeff's Van Gieson (EVG) staining kit (BA4083A, Baso, Zhuhai, China).

### Immunohistochemical (IHC) and immunofluorescence (IF) assay

To prepare for IHC staining, tissue sections underwent pre-dewaxing and hydration processes. Antigen retrieval was conducted utilizing a microwave technique in a sodium citrate buffer solution at a concentration of 0.01 mol/L (C0010, Solarbio, Beijing, China). This process involved sequential heating intervals of 2 minutes, 20 seconds, 10 seconds, and another 10 seconds, with cooling phases in between each heating session. Following this, the tissue was treated with 3% H₂O₂ (88,597, Sigma-Aldrich, MO, USA) for a duration of 15 minutes while being shielded from light. This was succeeded by a blocking procedure with goat serum (AR0009, Boster, Wuhan, China) for one hour. For IF, LF cells were fixed in 4% paraformaldehyde (30 min, RT) and permeabilized with Triton X-100 (T8787, Sigma-Aldrich) after blocking. 

Both tissues and cells were probed overnight at 4°C with primary antibodies: CILP (Abmart, PC13954S; 1:100), TGF-β1 (Affinity, AF1027; 1:100), COL1A2 (Affinity, DF3549; 1:100), α-SMA (Affinity, AF1032; 1:100), p-SMAD3 (Affinity, AF3362; 1:100), Vimentin (Affinity, DF7035; 1:200), and SERPINE2 (Abcam, ab154591; 1:200). Subsequently, IHC staining was conducted utilizing a goat anti- Rabbit IgG (H + L) HRP-conjugated Secondary antibody (Abcam, ab205718; 1:200). For IF detection, we employed FITC-conjugated (Abcam, ab6717; 1:500) and Cy3-conjugated secondary antibodies (Abcam, ab6939; 1:500). IHC visualization was achieved using a DAB substrate kit (Abcam ab64238), while nuclear counterstaining in IF utilized DAPI (Abcam, ab104139).

### Animal experiment

The development of the LFH mouse model in this study builds upon prior research findings. For this study, a total of 42 C57BL/6 male mice, each eight weeks old, were obtained from the Guangdong Experimental Animal Center located in Guangdong, China. The AAV2 vector used in this research was sourced from Hanbio Biotechnology Co., Ltd based in Shanghai, China.

Following a week of acclimatization, the mice were systematically assigned to one of seven distinct groups for the purpose of the study. These groups included a control group, which served as a baseline for comparison, as well as several experimental groups: the AAV2-vector group, the OE-CILP group, the si-CILP group, the BS group, the BS+OE-CILP group, and the BS+si-CILP group. This random division into groups was essential to ensure that any observed effects could be attributed to the specific treatments administered, thereby enhancing the reliability and validity of the experimental results. Subsequent to anesthesia with isoflurane gas, mice in the OE-CILP and si-CILP groups underwent L5/6 LF exposure under a surgical microscope. A microinjector was employed to administer a 3 μL virus solution into the left side of the spine. The remaining groups received injections of a blank carrier and were allowed a recovery period of 3 weeks following surgery. Throughout a period of 12 weeks, the mice belonging to the BS group were placed in a plastic mold where the water level was kept at 5 mm at the bottom. The study protocol involved inducing a bipedal standing posture for a total of 6 h daily, divided into two sessions with a two-hour break to allow free access to food and water, ensuring compliance with ethical animal care standards. Following this, the mice were sacrificed, and entire L5/6 spinal segments were collected for later histological and IHC staining examinations. Simultaneously, serum samples were collected from mice via cardiac blood sampling, temporarily stored in 1.5 mL Eppendorf tubes, and subsequently processed in accordance with the procedure outlined in Section 2.4 to obtain serum.

### Enzyme-linked immunosorbent assay (ELISA)

The liquid above the cultured cells was gathered, and the levels of human CILP and Collagen I were measured with ELISA kits (JM-6319 and JM-03329, respectively; Jingmei, Yancheng, China). Additionally, species-specific ELISA kits were employed to determine the concentration of CILP in mouse (JM-13217, Jingmei) serum samples.

### Statistical analysis

The data is presented as mean ± standard deviation (S.D.) and analyzed using SPSS software (version 25.0, IBM SPSS Statistics, NY, USA). Graphical illustrations were generated with GraphPad Prism software (version 7.1.0, GraphPad Software, CA, USA). When suitable, either Student's t-test or one-way ANOVA was applied, following preliminary checks for normality and variance homogeneity. A statistical significance level was set at *P*< 0.05.

## Results

### Transcriptomic analyses and iTRAQ results suggest differential expression of proteins and fibrotic nature in LFH

**Fig. 1:**
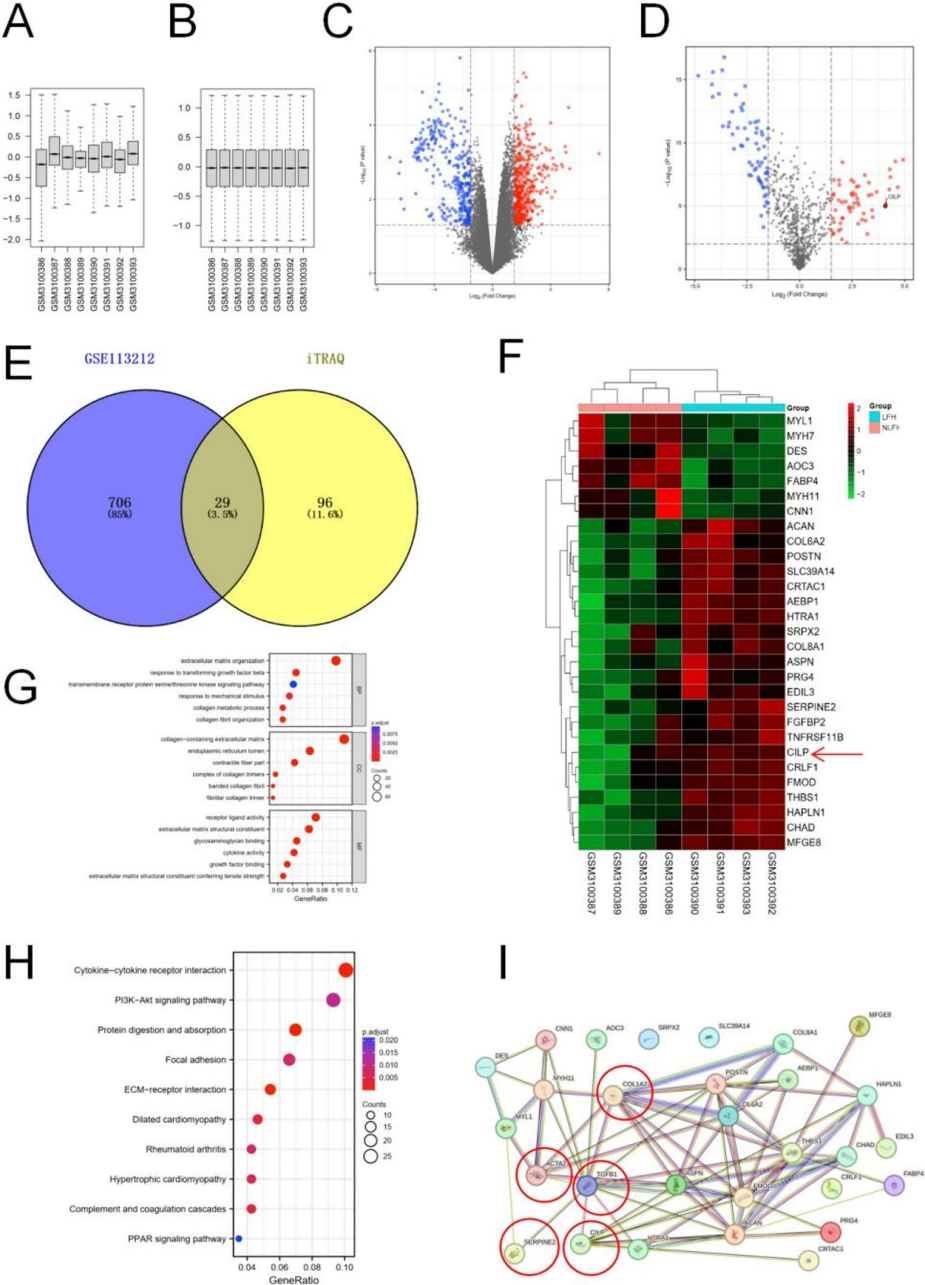
OGPs identification in LFH via Integrated Transcriptomic, Proteomic, and Bioinformatics Analyses. (**A**) LFH microarray dataset GSE113212 boxplot pre-standardization. (**B**) Standardized LFH microarray dataset GSE113212 boxplot. (**C**) Volcano plot showing 735 DEGs in GSE113212 from transcriptomic analysis. (**D**) Volcano plot displaying 125 DEPs identified in proteomic analysis. (**E**) Venn diagrams showing 29 OGPs. Blue section: 735 DEGs dataset, yellow section: 125 DEPs dataset. (**F**) Heatmap of the 29 OGPs in Non-LFH and LFH samples. (**G**) KEGG analysis of the overlapping genes. (**H**) GO analysis of the overlapping genes. (**I**) STRING protein-protein interaction network, highlighting key interactions involving CILP, TGF-β1, COL1A2, and α-SMA in a red square

We obtained the GSE113212 dataset related to LFH from the NCBI Gene Expression Omnibus. In order to assess the consistency of the data, we standardized the expression data from the LFH microarray dataset GSE113212 (Figure 1A-B). Subsequently, based on the criteria of P value < 0.05 and |log₂ fold change (FC)| > 1.5, 735 differentially expressed genes (DEGs) were identified and presented in the volcano plot, among which 429 were upregulated and 306 were downregulated (Figure 1C). In this study, iTRAQ analysis technology was used to obtain proteomic data from ligamentum flavum tissue. Based on the criteria of P value< 0.01 and |log2 fold change (FC)| > 1.5, a total of 125 differentially expressed proteins (DEPs) were identified, with statistical significance. Among these DEPs, 59 were upregulated and 66 were downregulated. (Figure 1D). The intersection of 735 DEGs and 125 DEPs was used to obtain 29 overlapping genes/proteins (OGPs), including 22 up-regulated and 7 downregulated genes (Figure 1E). These 29 OGPs were represented through heat maps (Figure 1F).

Notably, the CILP expression level was markedly elevated in the LFH group compared to the non-LFH group. Additionally, the heat map analysis revealed a significant upregulation of the CILP expression levels. Following that, GO Enrichment Analysis was performed on the OGPs to forecast their biological roles. The examination indicated that the OGPs were mainly linked to the Molecular Function (MF) categories, including ECM structural constituents, ECM structural constituent conferring tensile strength, growth factor binding, and glycosaminoglycan binding. The biological processes (BP) linked to OGPs mainly include ECM organization, response to transforming growth factor beta, response to mechanical stimulus, collagen metabolic process, and collagen fibril organization. Meanwhile, the cellular component (CC) type OGPs primarily involve collagen-containing ECM, contractile fiber part, complex of collagen trimers, banded collagen fibril and fibrillar collagen trimer. (Figure 1G).

The KEGG bubble plot delineates the associations between proteins and enrichment pathways (Figure 1H). The KEGG pathway enrichment analysis showed that the OGPs were mainly enriched in ECM-receptor interaction, Focal adhesion, Hypertrophic cardiomyopathy, and Protein digestion and absorption, and all these pathways are associated with fibrosis.

The interactions between CILP and genes including TGF-β1, α-SMA, and Collagen I were elucidated using the STRING tool. Furthermore, an interaction between SERPINE2 and TGF-β1 was identified (Figure 1I).

### scRNA-seq analysis of LF suggest differential expression of proteins and fibrotic nature in LFH

**Fig. 2:**
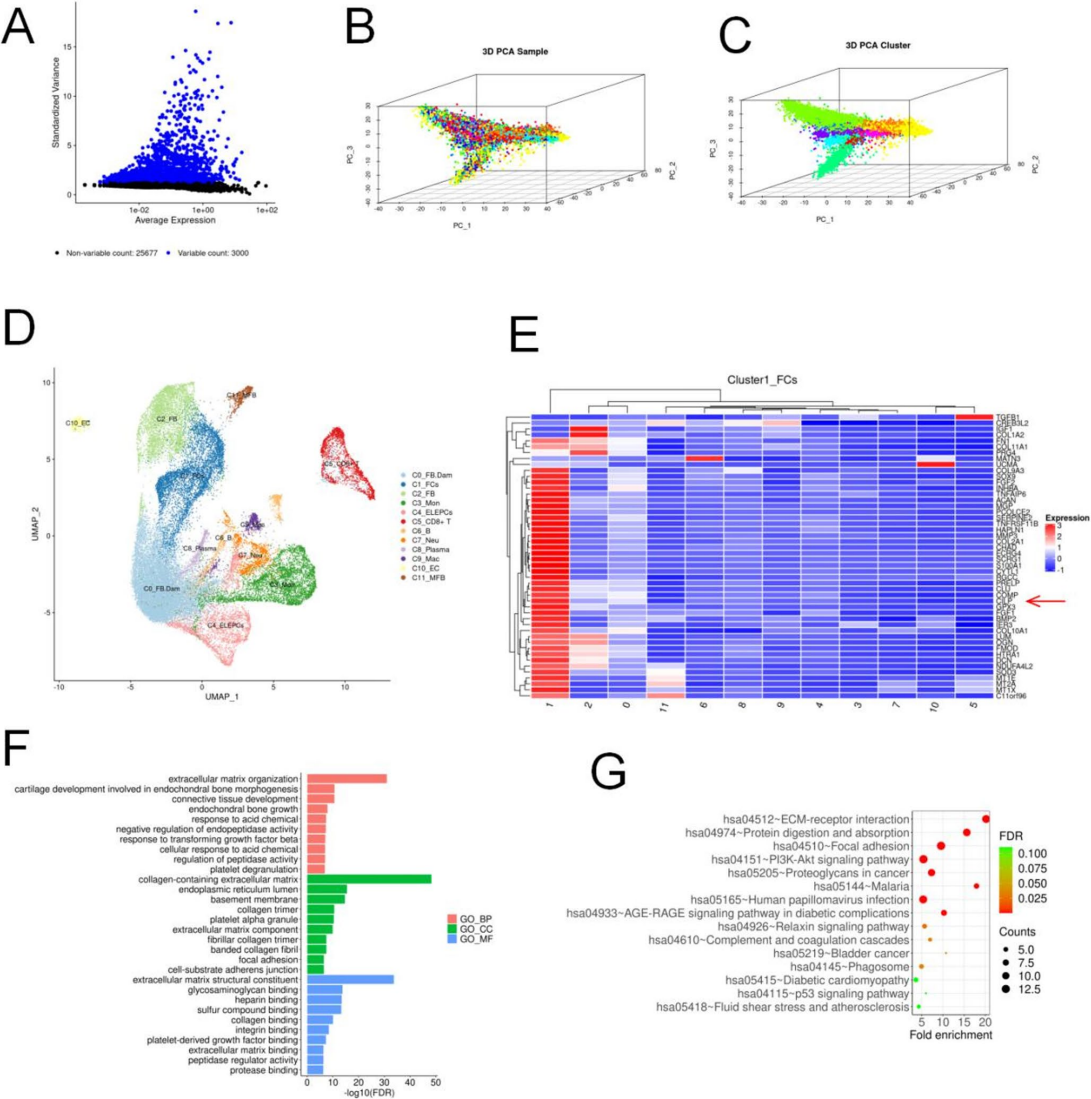
scRNA-seq analysis of the LF revealed elevated CILP expression in the LFH group. (**A**) Top 3,000 highly variable genes were selected for analysis. (**B**) 3D PCA visualization of clustering (grouped by sample). (**C**) 3D PCA visualization of clustering (grouped by cell type). (**D**) UMAP visualization of LF cells subpopulations highlighting differences. (**E**) Marker gene CILP was highly expressed in LF cells. (**F**) GO analysis of DEGs from scRNA-seq analysis. (**G**) KEGG analysis of DEGs from scRNA-seq analysis

We conducted scRNA-seq analysis of the LF tissue, employing various data visualization and analytical methods to explore cellular heterogeneity and function. We commenced our analysis by identifying differentially variable genes, which revealed 3,000 highly variable genes potentially associated with specific cellular functions or states, alongside 25,677 genes with low variability likely related to basic cellular functions (Figure 2A). We then utilized three-dimensional PCA to visualize the gene expression differences among samples, where distinct sample groupings were evident in the PC space, indicating sample similarities and differences (Figure 2B). To further investigate cellular heterogeneity, we performed PCA based on cell type, which clustered cells into distinct groups, potentially corresponding to different cell types or states, providing a foundation for subsequent biological analysis (Figure 2C). We proceeded with Uniform Manifold Approximation and Projection (UMAP) and identified 12 distinct cell groups, indicating a high level of cellular heterogeneity within the sample (Figure 2D). The 12 distinct cell groups included C0_FB.dam (fibros.damaged), C1_FCs (Fibrochondrocytes), C2_FB (fibroblasts), C3_Mon (monocytes), C4_ELEPCs (erythroid-like and erythroid precursor cells), C5_CD8+T (CD8+ T cells), C6_B (B cells), C7_Neu (neutrophils), C8_Plasma (plasma cells), C9_Mac (macrophages), C10_EC (endothelial cells), and C11_MFB (myofibroblasts). To gain insights into the gene expression characteristics of specific cell subgroups, a heatmap of gene expression was generated, highlighting the high expression of the CILP gene in Fibrochondrocytes (Figure 2E). GO analysis revealed that the DEGs were enriched in 10 biological processes, 10 cellular components, and 10 molecular functions. The most enriched protein was fibrosis-related biological behaviour (Figure 2F). The KEGG analysis revealed that Differentially expressed genes were involved in pathways related to fibrosis, including the ECM-receptor interaction and the focal adhesion pathway (Figure 2G).

### A high correlation exists between the activation of CILP and TGF-β1 signals and LF fibrosis in LFH tissues

**Fig. 3:**
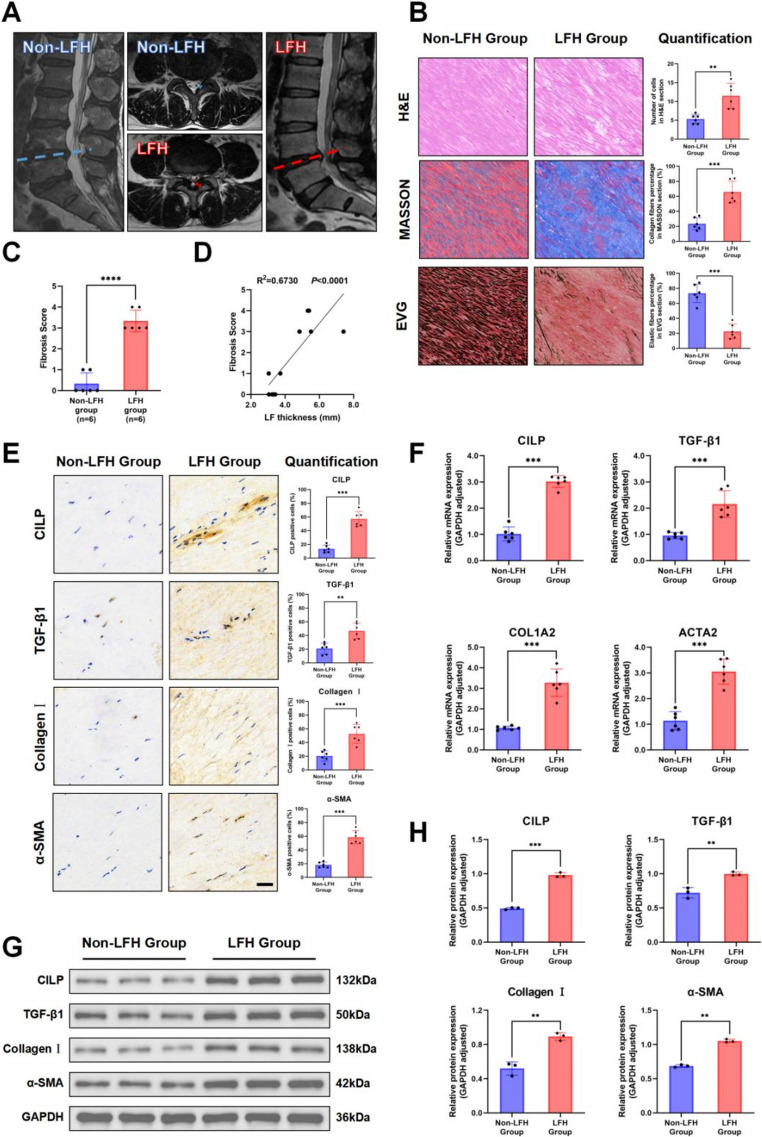
Fibrosis phenotype and the expression of CILP/TGF-β1 were markedly elevated. (**A**) Dashed lines delineate the middle layer of the L4/5 disc, while solid lines indicate the LF thickness. Blue coloration signifies normal LF, whereas red denotes LFH. (**B**) Histological sections from Non-LFH and LFH groups (n = 6 per group) groups were assessed using hematoxylin & eosin (H&E), Masson’s trichrome, and Verhoeff–Van Gieson (EVG) staining, scale bar: 50 μm. (**C**) Fibrosis scores in Non-LFH and LFH groups (n = 6 per group). (**D**) Fibrosis severity showed a direct linear relationship with LF thickening (R² = 0.6730, *P*< 0.0001). (**E**) IHC staining for CILP, TGF-β1, Collagen Ⅰ, and α-SMA in Non-LFH and LFH groups (n = 6 per group), with cell quantification. Scale bar: 50 μm. (**F**) qRT-PCR showed elevated CILP, TGF-β1, ACTA2, COL1A2 mRNA in Non-LFH and LFH groups (n = 6 per group). (**G**) Western blotting for CILP, TGF-β1, α-SMA and Collagen Ⅰ in Non-LFH and LFH groups (n = 6 per group), with GAPDH as loading control. (**H**) Quantified relative protein expression from WB. Significance levels are denoted as follows: **P< 0.01;***P< 0.001;****P< 0.0001

MRI was utilized to evaluate the LF thickness (Figure 3A). The results showed that individuals with LFH had an average LF thickness of 5.72 mm ± 0.26 mm, which was notably higher than the average thickness of 3.14 mm ± 0.11 mm recorded in the non-LFH individuals (Table 1).

The LF clinical samples were collected and subjected to analysis using histological staining, Western blotting, and qRT-PCR. The findings revealed that staining with H&E, EVG, and Masson's trichrome demonstrated an irregular, fragmented, and diminished arrangement of elastic fibers in hypertrophic LF, accompanied by a notable elevation in the proportion of collagen fibers. In the group without thickening, elastic fibers are uniformly distributed and relatively abundant, whereas the amount of collagen fibers is notably lower (Figure 3B). Meanwhile, the fibrosis score for LFH patients was 3.33 ± 0.21, markedly exceeding that of the LDH group (Figure 3C). Correlation analysis reveals a statistically significant linear regression relationship between fibrosis score and LFH thickness (Figure 3D).

In addition, in the LFH group, results from IHC staining (Figure 3E), qRT-PCR (Figure 3F), and Western blotting (Figure 3G) demonstrated significant upregulation in the expression levels of CILP, TGF-β1, and fibrosis markers Collagen I and α-SMA.

### TGF-β1 stimulated the expression of CILP and promoted fibrosis in LF cells in a time-dependent manner

**Fig. 4:**
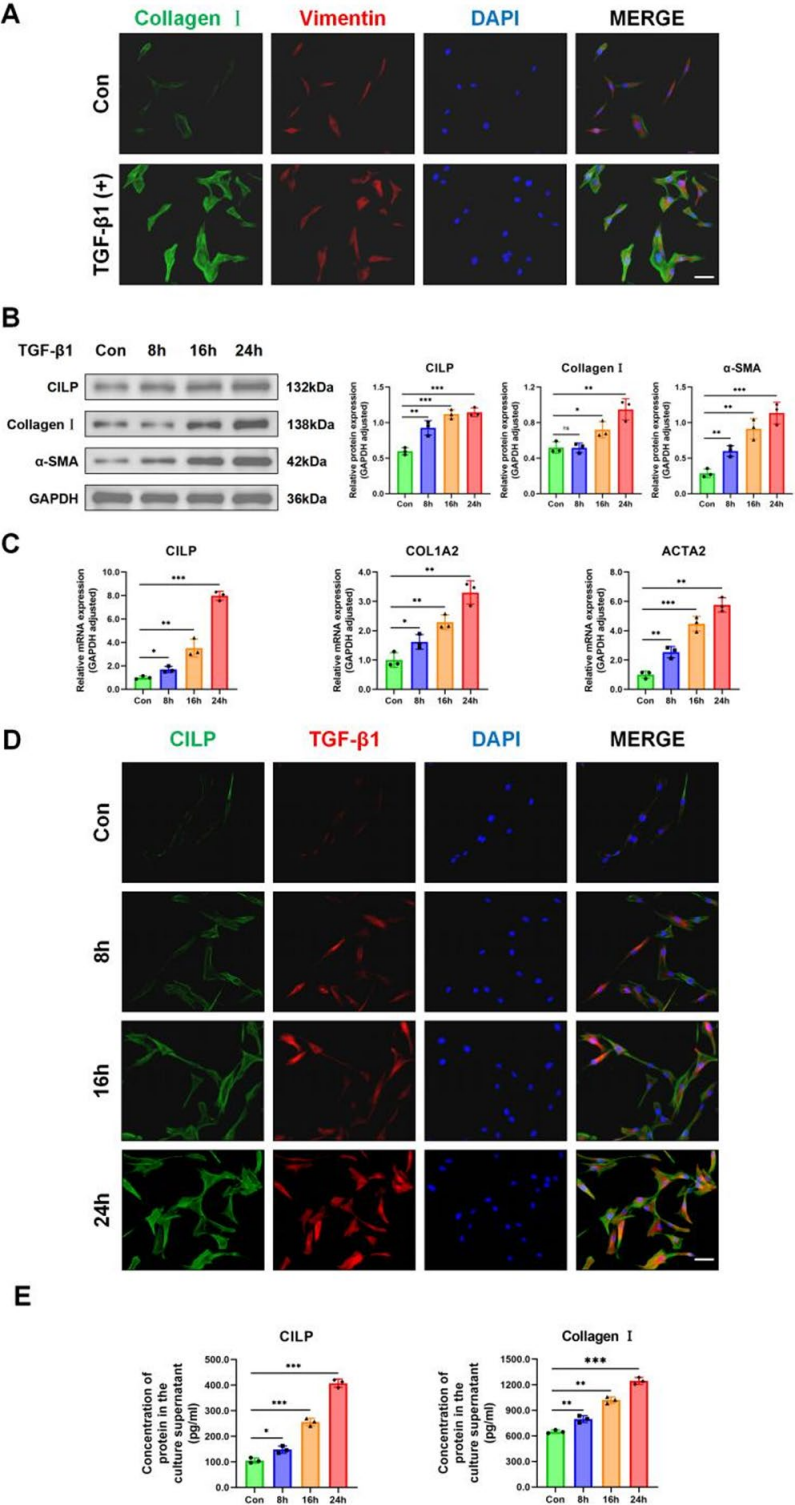
TGF-β1 induces fibrosis and enhances CILP expression in LF cells. (**A**) Double-labeling immunofluorescence was employed to stain Collagen I and vimentin. Nuclei were highlighted with DAPI (blue), Collagen Ⅰ appeared green, and vimentin was marked red. Scale bar: 20 µm. (**B**) Western blotting was conducted to evaluate the protein levels of CILP, α-SMA, and Collagen Ⅰ in LF cells stimulated by TGF-β1 at 0, 8, 16, and 24 h time points, and relative protein expression were quantified. (**C**) qRT-PCR analysis was performed to measure mRNA concentration of CILP, ACTA2 (α-SMA), and COL1A2 in LF cells treated with TGF-β1 at 0, 8, 16, and 24 h time points. (**D**) IF showed TGF-β1 and CILP after TGF-β1 treatment at 0, 8, 16, and 24 h time points. Nuclei stained blue with DAPI, CILP in green, and TGF-β1 in red. Scale bar: 20 µm. (**E**) ELISA assays determined CILP and Collagen Ⅰ protein expression levels in culture supernatant post-TGF-β1 induction at specified time points (0, 8, 16, and 24 h). Significance levels are denoted as follows: ns, not significant;*P< 0.05;**P< 0.01

The TGF-β1 protein may promote cellular fibrosis; nonetheless, its effect on CILP expression in LF cells is still unclear. Upon exposure to TGF-β1, LF cells exhibited elevated fluorescence expression of Collagen I and Vimentin, suggesting a transformation into myofibroblasts (Figure 4A). Furthermore, following treatment with TGF-β1 for 8, 16, and 24 h, there was an upregulation in the expression of CILP and fibrosis markers in LF cells (Figure 4B). In a similar manner, the qRT-PCR analysis illustrated an upward trend in the levels of CILP, COL1A1, and ACTA2 following prolonged exposure to TGF-β1 (Figure 4C). These findings were corroborated by IF assays (Figure 4D). Furthermore, ELISA results revealed the presence of secreted CILP and Collagen I in the culture supernatant following TGF-β1 treatment (Figure 4E).

 These findings illustrate that the increase in CILP expression and the fibrotic reaction in LF cells triggered by TGF-β1 relies on how long the exposure lasts.

### CILP inhibits TGF-β1-induced SMAD3 signaling and inhibits LFH

**Fig. 5:**
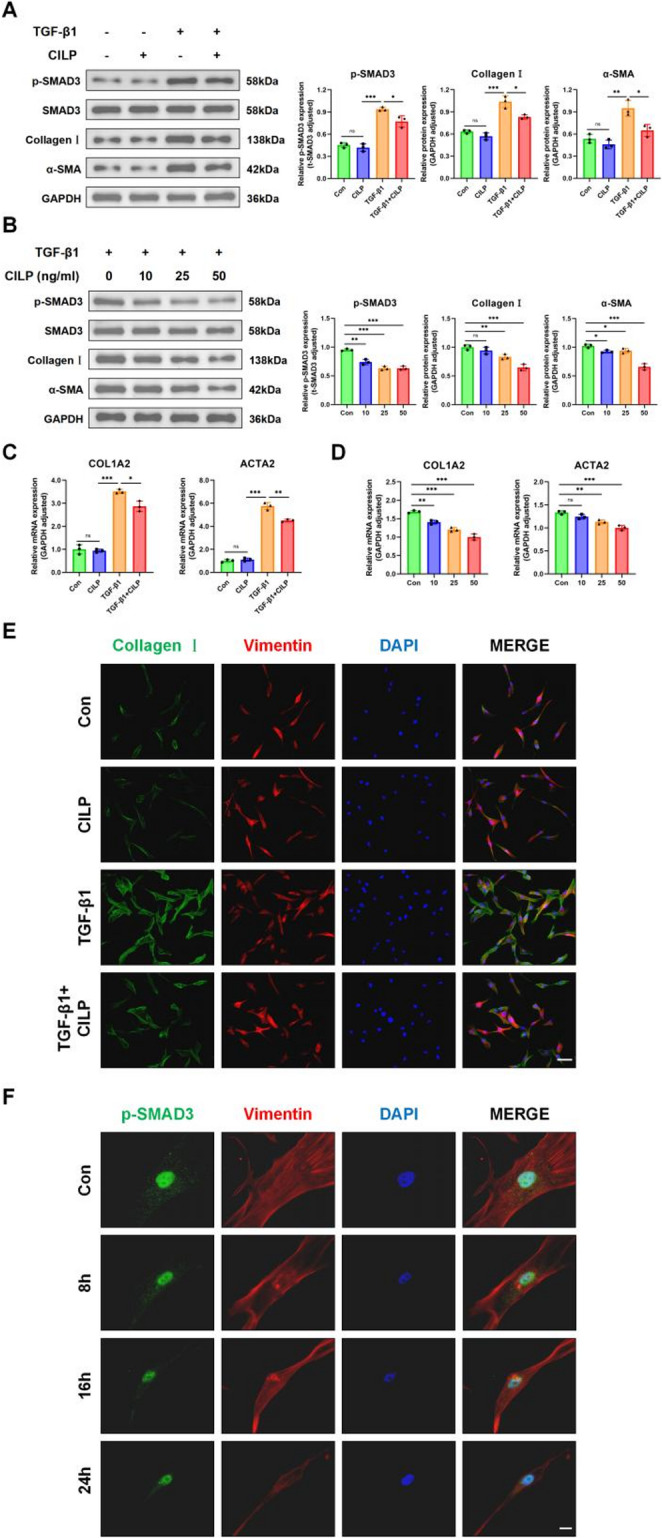
CILP inhibits TGF-β1-induced SMAD3 signaling. (**A**) After 24 hours of different treatments, p-SMAD3 and fibrosis-related protein levels in LF cells were evaluated. (**B**) Examination of p-SMAD3 and fibrotic protein expression in LF cells treated with TGF-β1 and varying concentrations of CILP for 24 hours. (**C**, **D**) qRT-PCR analysis determined COL1A2 and ACTA2 (α-SMA) gene expression levels in LF cells under varied treatments. (**E**) Double-labeling IF staining visualized Collagen I and Vimentin in LF cells post 24-hour treatments. Nuclei stained blue with DAPI, Collagen Ⅰ in green, and Vimentin in red. Scale bar: 20 µm. (**F**) Confocal microscopy demonstrated that CILP attenuates TGF-β1-induced enhancement of p-SMAD3 nuclear localization in LF cells. Cells treated with TGF-β1 for 24 hours and CILP for 0, 8, 16, and 24 hours. Nuclei stained blue with DAPI, p-SMAD3 in green, and Vimentin in red. Scale bar: 10 µm. Significance levels are denoted as follows: ns, not significant; *P< 0.05; **P< 0.01; ***P< 0.01

Earlier research has indicated that the TGF-β1/SMAD3 signaling pathway is significant in the fibrosis of the LF; however, the mechanism by which CILP influences this process remains ambiguous. The findings indicated that incorporating CILP results in a notable decrease in SMAD3 phosphorylation by TGF-β1 protein activation. Additionally, there was a decrease in the expression levels of Collagen I and α-SMA (Figure 5A). Subsequent WB analyses demonstrated that increasing concentrations of CILP protein progressively augmented the inhibition of TGF-β1-induced thickening of the LF (Figures 5B). At the same time, the above results were confirmed by qRT-PCR results (Figure 5C, D). Additionally, IF assays revealed that CILP markedly suppressed the upregulated expression of Collagen I and Vimentin proteins associated with LF fibrosis (Figure 5E).

The process of phosphorylating SMAD3, along with its later movement into the nucleus, plays a vital role in TGF-β1 signaling pathways. To clarify the effect of CILP on phosphorylated SMAD3 and its movement into the nucleus, IF studies demonstrated that treatment with TGF-β1 significantly increased the levels of nuclear p-SMAD3. Conversely, the exogenous application of CILP significantly impeded the nuclear transport of SMAD3 (Figure 5F). The results suggest that external CILP could operate by blocking the SMAD3 signaling pathways activated by TGF-β1 and reducing the transcriptional activity driven by TGF-β1.

### CILP has the potential to attenuate fibrotic lesions in the LF by modulating the feedback mechanism of the TGF-β1/SMAD3 and SERPINE2 pathways

**Fig. 6:**
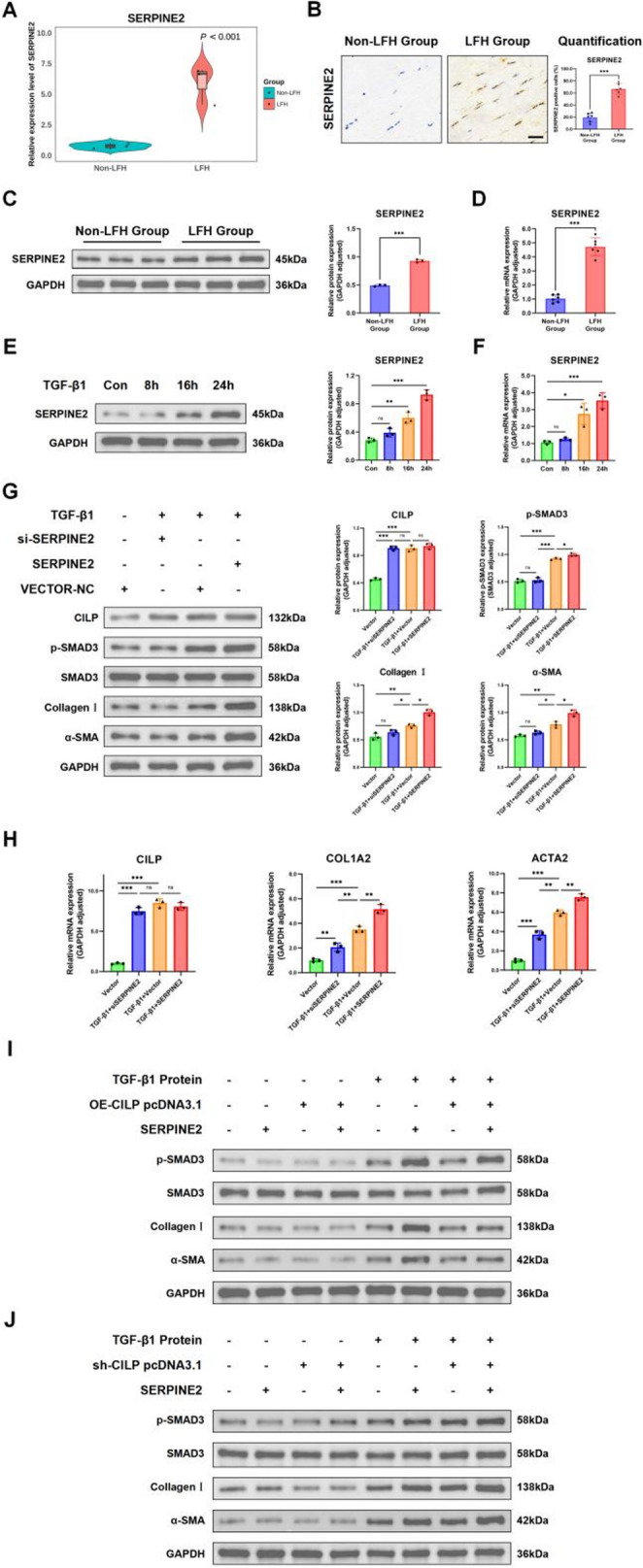
CILP attenuates LF fibrosis via the TGF-β1/SMAD3/SERPINE2 axis. (**A**) A violin plot depicted differential SERPINE2 expression between Non-LFH and LFH groups (n = 6 per group), showing significant differences. (**B**) IHC of SERPINE2 in LF samples from Non-LFH and LFH groups (n = 6 per group). Scale bar: 50 µm. (**C**, **D**) Western blotting revealed elevated SERPINE2 protein levels between Non-LFH and LFH groups (n = 6 per group), with relative protein expression quantified. And mRNA concentration showed consistent results. (**E**, **F**) Western blotting demonstrated SERPINE2 expression in LF cells treated with TGF-β1 at 0, 8, 16, and 24 h time points, with relative protein expression quantified. And mRNA concentration showed consistent results. (**G**) Western blotting assessed protein expression levels of CILP, Collagen I, p-SMAD3, and α-SMA in LF cells subjected to various SERPINE2 treatments. (**H**) qRT-PCR assessed transcriptional profiles of CILP, ACTA2 (α-SMA), and COL1A2 across experimental conditions in LF cells. (**I**) WB analysis evaluated p-SMAD3 and fibrotic protein levels 48 hours post-transfection with CILP-overexpressing plasmids under different conditions in LF cells. (**J**) WB analysis examined p-SMAD3 and fibrotic protein expression 48 hours post-transfection with CILP-silencing plasmids under various regimens. Significance levels are denoted as follows: ns, not significant;*P< 0.05;**P< 0.01;***P< 0.01

SERPINE2, a crucial protein, regulates the degradation of the ECM and could greatly influence the formation of fibrotic lesions linked to LFH. The iTRAQ analysis illustrated a notable upward trend in the expression levels of SERPINE2 within the LFH cohort (Figure 6A). Consistent findings were observed through IHC, qRT-PCR, and WB analyses (Figure 6B-D). In addition, similar to the findings observed in human samples, SERPINE2 mRNA and protein expression levels were higher in the* in vitro* cell model (Figure 6 E-F). This indicates that SERPINE2 could be crucial in the development of LFH.

To clarify the function of SERPINE2 in LF cell fibrosis, we utilized siRNA to reduce the SERPINE2 expression in these cells. WB analysis revealed that siRNA transfection led to a marked reduction in SERPINE2 expression levels. This downregulation was accompanied by decreased phosphorylation of SMAD3 and reduced levels of fibrosis-related proteins. Notably, the expression level of CILP remained unchanged. Conversely, overexpression of SERPINE2 via a plasmid resulted in increased levels of phosphorylated SMAD3 and fibrosis-associated proteins, yet it did not alter CILP expression. These protein-level alterations were further supported by qRT-PCR analysis of corresponding mRNA expression patterns (Figure 6G).

To provide a clearer understanding of how CILP functions within the TGF-β1/SMAD3 and SERPINE2 signaling pathways, plasmids for both overexpressing and interfering with human CILP were utilized in the treatment of LF cells. Consistent with prior findings, CILP overexpression effectively attenuated p-SMAD3 and fibrotic protein expression. However, the concurrent presence of CILP and SERPINE2 impeded subsequent phosphorylation and fibrotic processes, as demonstrated by the CILP interference results (Figure 6I). These observations suggest that CILP may modulate the feedback mechanism between TGF-β1/SMAD3 and SERPINE2 in LFH lesions.

### CILP reduces LFH *in vivo* mice model through the inhibition of the TGF-β1/SMAD3/SERPINE2 signaling pathway

**Fig. 7:**
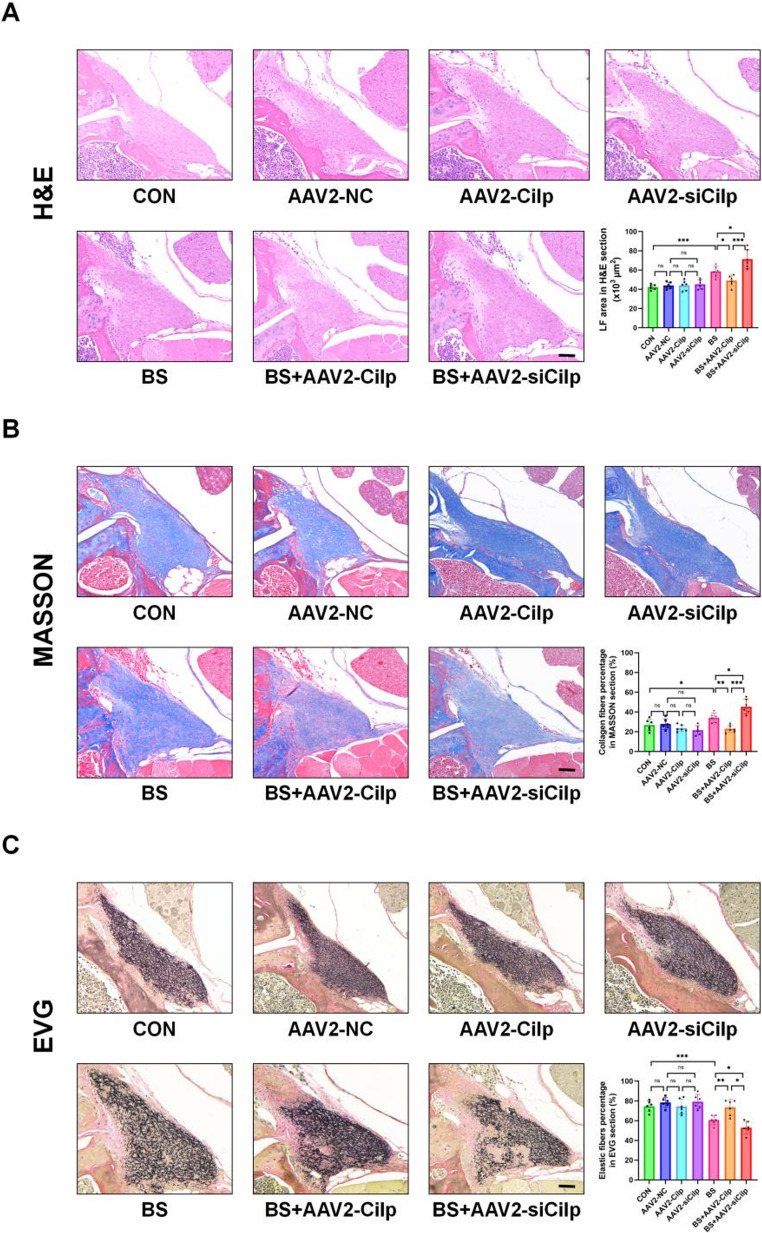
Fibrosis was significantly exacerbated in the LFH group in vivo. (**A**) Histological assessment via H&E was performed on mouse tissues (n = 6 each group), followed by quantitative measurement of LF cross-sectional area. Scale bar: 50 µm. (**B**) Histological assessment via MASSON of mouse tissues was conducted, followed by quantitative analysis of the collagen fiber percentage in LF. Scale bar: 50 µm. (**C**) Histological assessment via EVG staining of mouse tissues was carried out, with quantitative analysis of the elastin fiber percentage in LF. Scale bar: 50 µm. Significance levels are denoted as follows: ns, not significant;*P< 0.05; **P< 0.01; ***P< 0.001

The previously mentioned *in vitro* studies indicate that TGF-β1 can trigger the induction of CILP, which then appears to provide a negative feedback inhibition on the TGF-β1/SMAD3/SERPINE2 signaling pathway, while also suppressing LFH fibrosis. Consequently, it is imperative to evaluate the potential of CILP to inhibit LFH in animal models. Histological examination revealed that, when compared to the CON group, the average LF area in BS mice showed a significant increase after a duration of 12 weeks. In addition, when compared to the CON group, the LFH group showed a notable rise in the ratio of collagen fibers, while also showing a significant decrease in the amount of elastin fibers (Figure 7 A-C).

AAV-2 vectors were utilized for direct injection into the LF to enhance the understanding of CILP's function in TGF-β1-induced LFH. In comparison to the CON group, there were no notable differences found in the area of the LF, collagen fiber content, or elastin ratio when examining the OE-CILP group alongside the si-CILP group. However, notably, in comparison to the BS group, the si-CILP+ BS group exhibited more pronounced LFH lesions, characterized by an increased area of the LF, elevated collagen fiber content, and reduced elastic fiber content. On the contrary, the OE-CILP+ BS group significantly inhibited the typical pathological changes of LFH histologically. The histological results indicate that CILP can significantly inhibit LFH fibrosis lesions in animal models.

**Fig. 8:**
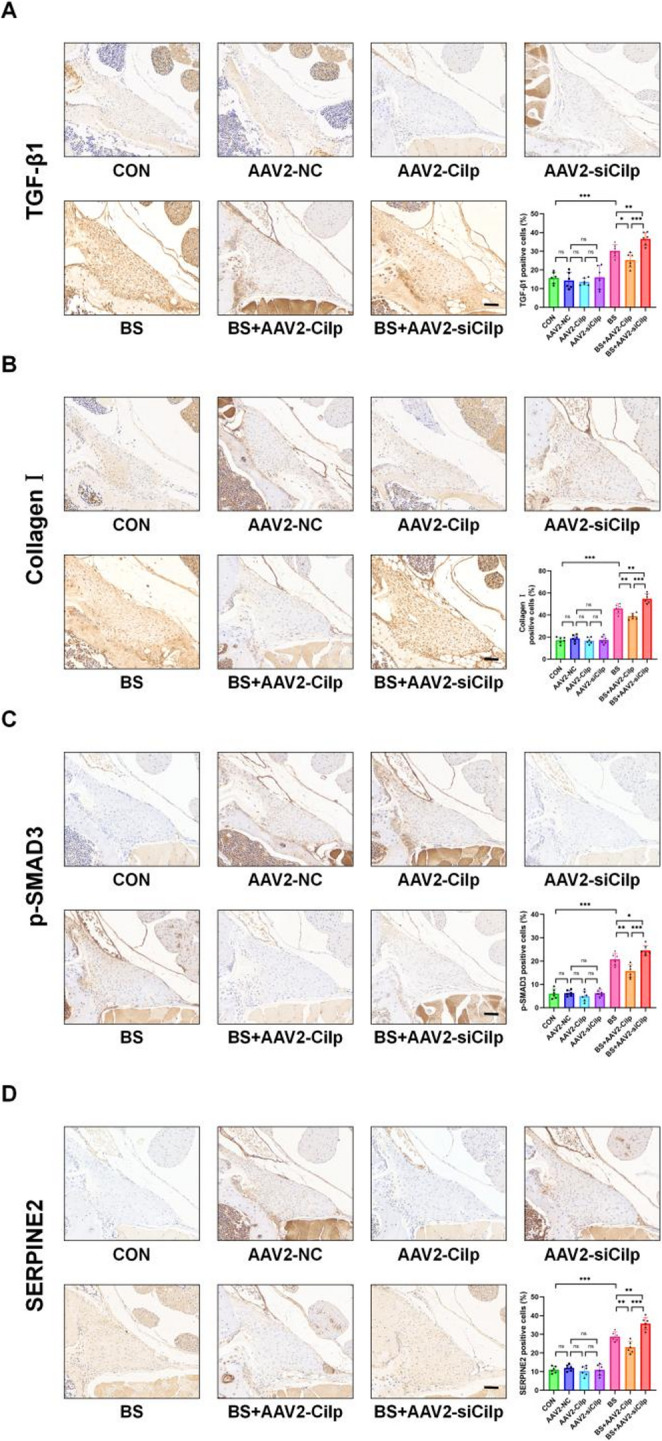
CILP attenuates TGF-β1-mediated LFH in vivo. (**A**-**D**) IHC analysis of SERPINE2, TGF-β1, Collagen I, and p-SMAD3 in mouse tissues, with quantification of positive cell percentages. Scale bar: 50 µm. Significance levels are denoted as follows: ns, not significant;*P< 0.05; **P< 0.01; ***P< 0.001

In addition, IHC staining was utilized to access the levels of TGF-β1 levels, SMAD3, SERPINE2, and markers associated with fibrosis after conducting experimental modeling and/or manipulating the expression of CILP through overexpression or knockdown. Consistent with earlier research results, no significant variations were detected in the expression levels of TGF-β1 and p-SMAD3 proteins across the CON group, the OE-CILP group, and the si-CILP group when BS modeling was not present. Following the reduction of CILP expression, the si-CILP+ BS group showed an upward trend in the levels of TGF-β1 and p-SMAD3 when compared to the BS group. In contrast, the OE-CILP+ BS group displayed an opposing trend. Similar differences in expression levels were observed for Collagen I, α-SMA, and SERPINE2 (Figure 8).

### CILP is stable in peripheral blood among LFH patients and BS mice model

ELISA assays were performed on samples obtained from human patients with both non-LFH and LFH groups, as well as on mouse serum from animal studies, to assess peripheral blood outcomes. It is important to highlight that the differential expression of CILP was identified in LFH specimens and cells throughout both* in vitro* and* in vivo* animal studies; however, no such differential expression was found in the serum samples (Figure S1).

## Discussion

LFH is identified as the primary pathological factor contributing to LSS[23]. The etiology of LFH is multifactorial, with current hypotheses suggesting that local aberrant inflammatory responses[23], mechanical stress[8], and natural degenerative processes may all contribute to its development. The primary pathological change linked to LFH involves tissue fibrosis, leading to the irregular accumulation of the ECM and the transformation of fibroblasts into myofibroblasts in the LF[24]. The progressive deterioration of the local ECM environment facilitates the reduction or degradation of elastic fibers during cellular transformation, which is associated with a substantial accumulation of collagen fibers[25]. In this study, LF specimens were acquired via minimally invasive lumbar surgical techniques, such as UBE or PELD surgeries, and subsequently subjected to detailed fibrosis staining analysis[26]. The findings indicated that the LF samples derived from the LFH group exhibited a significantly greater thickness compared to those from the non-LFH group, along with a marked accumulation and degradation of collagen fibers. These indicated that the human LF samples collected in the experiment exhibit characteristic and significant differences in fibrosis.

Prior clinical and animal studies have demonstrated that the inflammatory response within the LF tissue, particularly at fibrotic lesions, significantly influences the onset, development, and progression of hypertrophic diseases of the LF[27, 28]. Consequently, we employed Transcriptomic analyses and iTRAQ detection to identify DEPs. Subsequent bioinformatics analysis revealed potential fibrotic abnormalities. Additionally, a notable elevation was observed in the expression levels of the CILP protein, which captured our attention. To further validate these findings, we employed scRNA-seq to examine CILP expression in normal and hypertrophic LF tissues. CILP was predominantly expressed in fibrochondrocytes. GO analysis of CILP revealed significant enrichment of fibrosis-related biological processes, while KEGG analysis identified associations with fibrotic pathways including ECM-receptor interaction and focal adhesion. These results demonstrate CILP's involvement in fibrotic processes and LFH. Notably, CILP expression was significantly upregulated in LFH compared to non- LFH groups, consistent with both transcriptomic and iTRAQ proteomic analyses. Further investigations employing IHC, Western blotting, and qRT-PCR supported the previously mentioned results. The findings indicate an unusual activation of CILP, TGF-β1, and a fibrotic phenotype in human LFH lesions, aligning with the findings from the bioinformatics analysis.

CILP, a non-collagenous protein predominantly expressed in cartilage tissue, is integral to the formation and maintenance of the ECM and is implicated in various degenerative diseases. This protein modulates interactions among diverse cellular signaling pathways, notably influencing the activity of TGF-β1. TGF-β1 serves as a powerful pro-fibrotic cytokine that is crucial in the development of fibrotic conditions, such as lung, heart, and kidney fibrosis[29–31]. Likewise, TGF-β1 encourages the growth and activation of LF cells, while also aiding in the development of myofibroblasts and the accumulation of ECM[23]. TGF-β1 binds to TGF-β1 receptor, facilitating receptor phosphorylation. This interaction subsequently activates the downstream SMAD3 protein, which forms a complex that translocates to the nucleus, where it exerts its functional role. *In vitro* cellular experiments have demonstrated that CILP can be activated by TGF-β1 stimulation, subsequently inhibiting SMAD3 phosphorylation and p-SMAD3 nuclear translocation. This process ultimately mitigates LFH lesions through a mechanism of negative feedback regulation. Therefore, this inhibitory effect establishes a negative feedback mechanism that hinders fibroblast proliferation and ECM deposition.

Subsequent investigations have highlighted the significance of the SERPINE2 protein. The SERPINE2 protein is integral to the fibrosis process through its involvement in ECM degradation, modulation of the inflammatory response, and activation of fibrosis signaling pathway[32–34]. Data derived from bioinformatics analyses and human specimens indicate a marked upregulation of the SERPINE2 protein in TGF-β1-induced LF fibrosis. Further investigation utilizing overexpression and knockout models in LF cells indicates that alterations in SERPINE2 expression may enhance the development of fibrotic lesions. Interestingly, these changes do not influence the expression levels of CILP. Conversely, CILP overexpression has been shown to inhibit SERPINE2 protein activity, whereas CILP suppression exacerbates the profibrotic effects of SERPINE2 through the TGF-β1/SMAD3 signaling pathway under identical conditions. Based on our current understanding, CILP may exhibit an antagonistic or inhibitory interaction with SERPINE2 in LFH lesions; however, the precise pathway underlying this relationship requires further investigation.

Drawing from previous research results, we utilized a BS mouse model to investigate the function of CILP in LFH[11, 35]. In comparison to New Zealand rabbit and rat models[36, 37], this model enables efficient and minimally invasive LFH modeling in mice. AAV2 demonstrates strong targeting capabilities in central nervous system and spinal research[38], allowing for stable overexpression or knockdown of target genes[39]. Histological examinations demonstrated that the *in situ* injection method can establish an LFH model while preserving the integrity of the existing structures. Furthermore, mice in the BS group exhibited more pronounced thickening of the LF. After injection with the overexpression of CILP vector, the histological results, TGF-β1/SMAD3/SERPINE2, and fibrosis markers of BS mice were significantly altered, while the interference vector could significantly alleviate LFH lesions. The findings suggest that CILP additionally suppresses LFH triggered by the TGF-β1/SMAD3/SERPINE2 signaling pathway in a living organism.

In alignment with findings from* in vitro* studies, it is important to highlight that the functions of CILP, both enhancing and attenuating, on LFH lesions are activated under BS conditions, rather than under typical unstimulated circumstances. This observation implies that CILP may function as a protein capable of preventing or potentially reversing LFH, thereby exhibiting both prophylactic and therapeutic properties in the context of LFH lesions. To further explore the diagnostic potential of CILP in LFH disease, we assessed peripheral CILP levels in both human and murine subjects. The findings revealed no statistically significant differences, indicating that while CILP may function locally within the LF as a therapeutic agent, it does not appear to serve as an effective systemic biomarker for the disease, unlike its role in cardiac and pulmonary fibrosis[40, 41]. Consequently, localized *in situ* administration of CILP protein may represent a more effective strategy for systemic intervention.

This study is subject to several limitations, although. Firstly, it exclusively examined the role of CILP within the TGF-β1/SMAD3 pathway. As a promising anti-fibrotic molecule, its potential involvement in LFH lesions via alternative pathways remains uncertain. Furthermore, the influence of CILP on SERPINE2 has not been elucidated, necessitating further molecular interaction studies. Additionally, the use of genetically modified mice, as opposed to surgical injection of the LF, could further minimize experimental interference in animal studies.

To summarize, this research highlights the significant inhibitory function of CILP in TGF-β1 induced LFH. Additionally, CILP exerts a negative influence on flavum ligament fibrosis via the TGF-β1/SMAD3/SERPINE2 signaling pathway.

## Supplementary Information

Below is the link to the electronic supplementary material.

Supplementary Material 1 (DOCX 18.1 KB)

Supplementary Material 2 (DOCX 16 KB)

Supplementary Material 3 (DOCX 3.68 MB)

## Data Availability

Data are available upon request from the corresponding author.
