# Supplementary material for: Cartilage intermediate layer protein inhibits ligamentum flavum hypertrophy mediated by TGF-β1/SMAD3/SERPINE2 signaling pathway
Source: Cell Mol Life Sci. 2026 Feb 9;83(1):108. doi: 10.1007/s00018-025-06051-7 (PMC12909686; doi:10.1007/s00018-025-06051-7)
Supplement: Supplementary file 1 — Supplementary Material 1 (DOCX 18.1 KB) [file 18_2025_6051_MOESM1_ESM.docx]

**Supplementary Table S1** Processing of each human LF sample in the research.

| **Total samples** | **Storage** | **Assays** |
| --- | --- | --- |
| 42 LF samples  (non-LFH  group: LFH  group = 21:21)  were obtained.  Three non-LFH  samples used for  iTRAQ assay  were evenly  divided into two  parts for sterile  cell culture. | 6 LF samples were used for iTRAQ assay | Isobaric tags for iTRAQ  (non-LFH group: LFH group =  3:3) |
|  | 12 LF samples were stored  in 4% paraformaldehyde | H&E, Masson's trichrome and EVG staining, Immunohistochemistry  (non-LFH group: LFH group =  6:6) |
|  | 24 LF samples were stored  in liquid nitrogen | qRT-PCR assay  (non-LFH group: LFH group =  6:6) |
|  |  | WB analysis  (non-LFH group: LFH group = 6:6) |
|  | 3 LF samples were used for cell culture | Cell culture  (non-LFH group: LFH group =  3:0) |
