# Supplementary material for: Cartilage intermediate layer protein inhibits ligamentum flavum hypertrophy mediated by TGF-β1/SMAD3/SERPINE2 signaling pathway
Source: Cell Mol Life Sci. 2026 Feb 9;83(1):108. doi: 10.1007/s00018-025-06051-7 (PMC12909686; doi:10.1007/s00018-025-06051-7)
Supplement: Supplementary file 2 — Supplementary Material 2 (DOCX 16 KB) [file 18_2025_6051_MOESM2_ESM.docx]

**Supplementary Table S2** Primer sequences and amplicon characteristics used for qRT-PCR analysis.

| **Gene** | **Forward primer (5′–3′)** | **Reverse primer (5′–3′)** | **Expected amplicon size (bp)** | **Annealing temperature (°C)** | **GenBank Accession No.** |
| --- | --- | --- | --- | --- | --- |
| **CILP** | **GCCCTGGTGAGTGGACAAC** | **GTCAGTGGTCCGAGCCTCTA** | **140** | **60** | **NM_003613.4** |
| **TGF-β1** | **CACGTGGAGCTGTACCAGAA** | **GAACCCGTTGATGTCCACTT** | **219** | **60** | **NM_000660.7** |
| **COL1A2** | **AAAGAACCCAGCTCGCA** | **GGATACAGGTTTCGCCAGT** | **146** | **60** | **NM_000089.4** |
| **ACTA2** | **CCTTGAGAAGAGTTACGAGTTG** | **TGCTGTTGTAGGTGGTTTCA** | **140** | **60** | **NM_001406467.1** |
| **SERPINE2** | **TGGTGATGAGATACGGCGTAA** | **GTTAGCCACTGTCACAATGTCTT** | **101** | **60** | **NM_001136528.2** |
| **GAPDH** | **CAATGACCCCTTCATTGACC** | **GACAAGCTTCCCGTTCTCAG** | **106** | **60** | **NM_001256799.3** |
