# Supplementary material for: Cartilage intermediate layer protein inhibits ligamentum flavum hypertrophy mediated by TGF-β1/SMAD3/SERPINE2 signaling pathway
Source: Cell Mol Life Sci. 2026 Feb 9;83(1):108. doi: 10.1007/s00018-025-06051-7 (PMC12909686; doi:10.1007/s00018-025-06051-7)

**Supplementary Figure S1**

Figure 3G

**Non-LFH Group LFH Group**


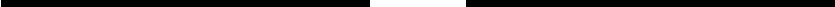


**CILP**
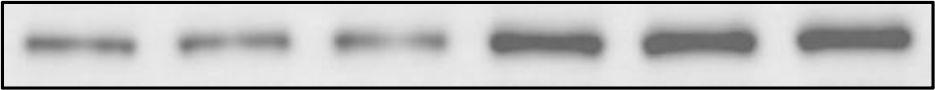
 **132kDa TGF-β1**
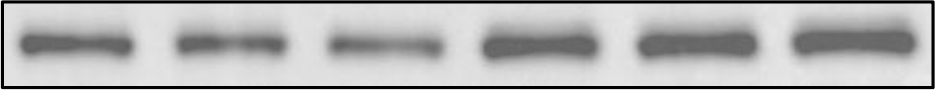
 **50kDa Collagen Ⅰ**
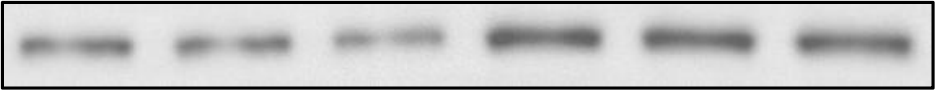
 **138kDa α-SMA**
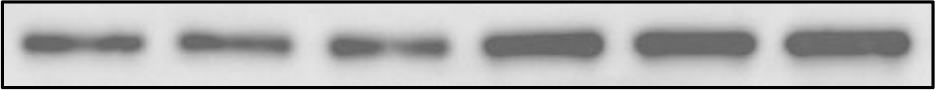
 **42kDa GAPDH**
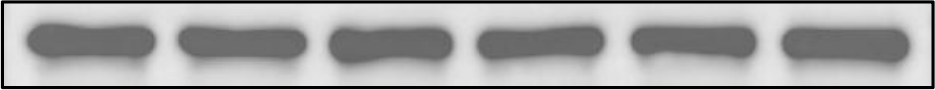
 **36kDa**

**CILP**

**132kDa**

**TGF-β1 50kDa**

**Collagen Ⅰ**

**138kDa**

**α-SMA**

**42kDa**

**GAPDH**

**36kDa**

**Non-LFH Group LFH Group**


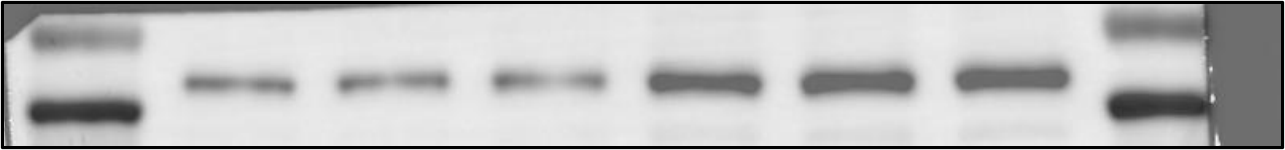

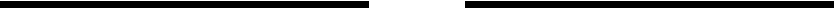


**150kDa**

**130kDa**


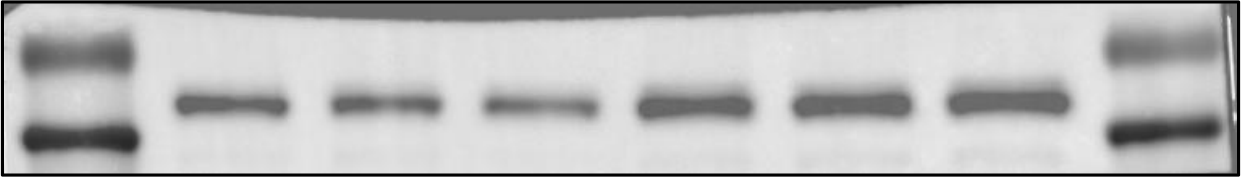


**55kDa**

**35kDa**


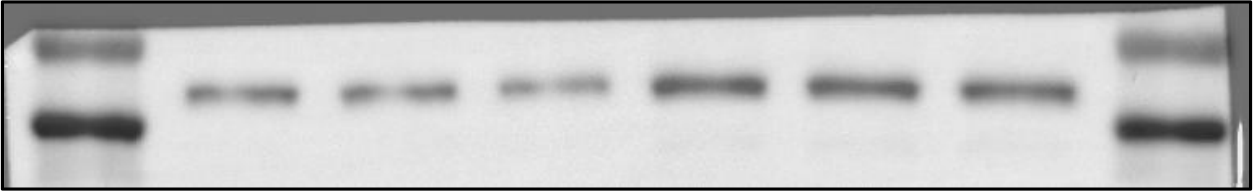


**150kDa**

**130kDa**


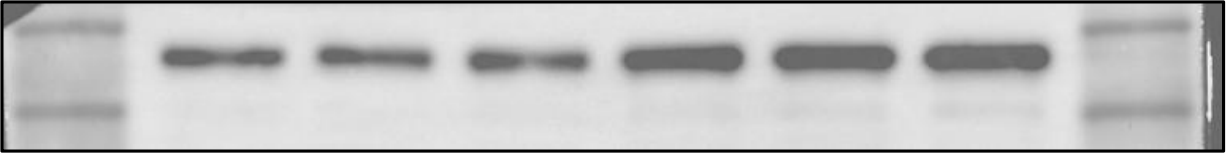


**55kDa**

**35kDa**


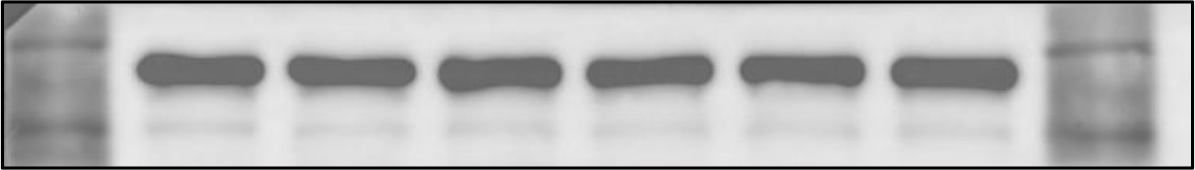


**55kDa**

**35kDa**

**Supplementary Figure S1**

Figure 4B

| **TGF-β1 CILP Collagen Ⅰ** | 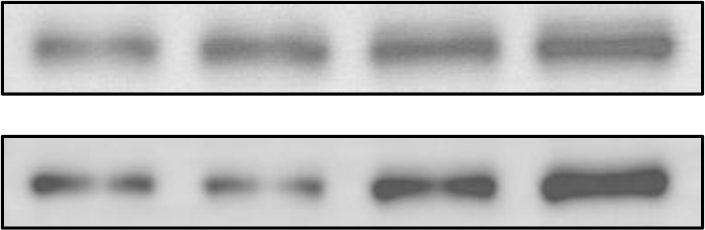**Con 8h 16h 24h**  **132kDa**  **138kDa** |
| --- | --- |

**α-SMA**
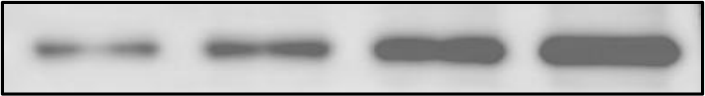
 **42kDa GAPDH**
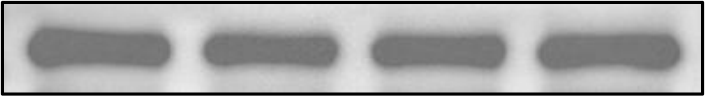
 **36kDa**


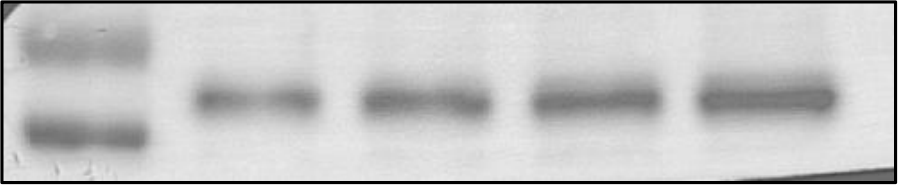
**TGF-β1 Con 8h 16h 24h**

**150kDa**

**130kDa**


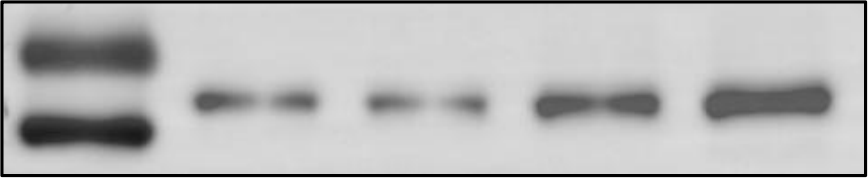


**150kDa**

**130kDa**


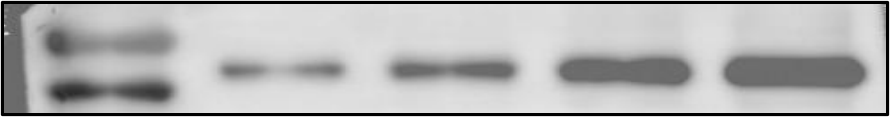
**55kDa**

**35kDa**


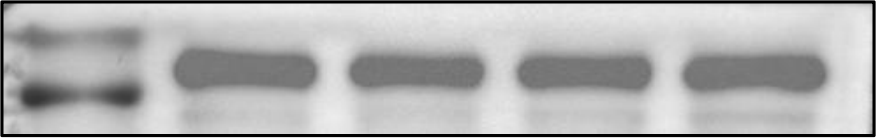


**55kDa**

**35kDa**

**CILP**

**132kDa**

**Collagen Ⅰ**

**138kDa**

**α-SMA**

**42kDa**

**GAPDH**

**36kDa**

**Supplementary Figure S1**

Figure 5A

**TGF-β1 CILP**

**+**

**+**

**+**

**-**

**-**

**+**

**-**

**-**

| **p-SMAD3**  **SMAD3**  **Collagen Ⅰ**  **α-SMA**  **GAPDH** | 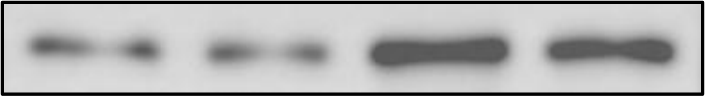  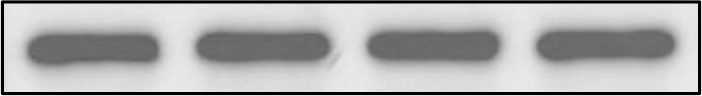  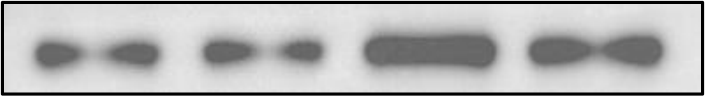  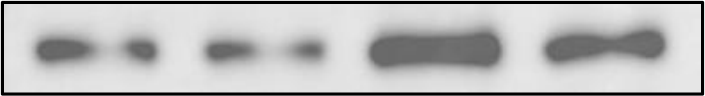  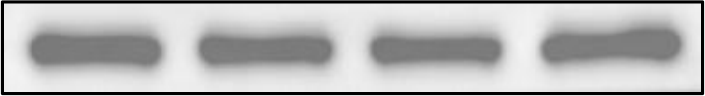 | **58kDa**  **58kDa**  **138kDa**  **42kDa**  **36kDa** |
| --- | --- | --- |

**70kDa**

**55kDa**

**70kDa**

**55kDa**

**150kDa**

**130kDa**

**55kDa**

**35kDa**

**55kDa**

**35kDa**

**TGF-β1 CILP**

**+**

**+**

**+**

**-**

**-**

**+**

**-**

**-**


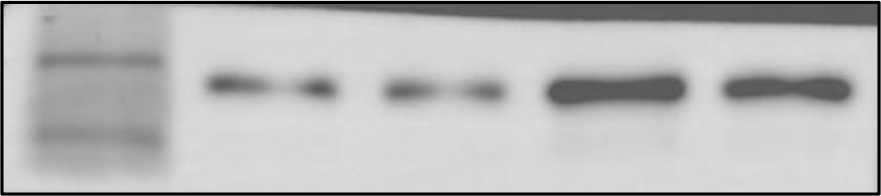


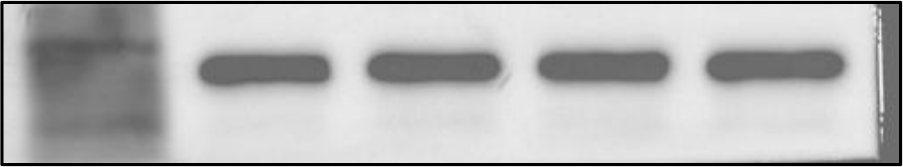


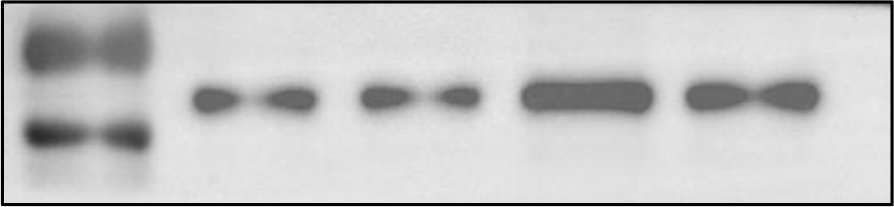


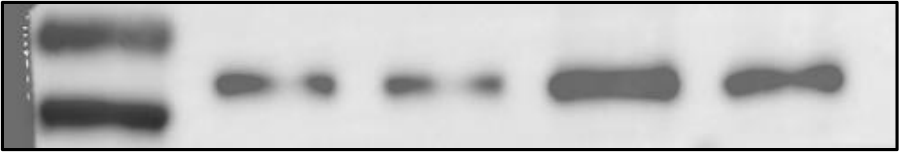


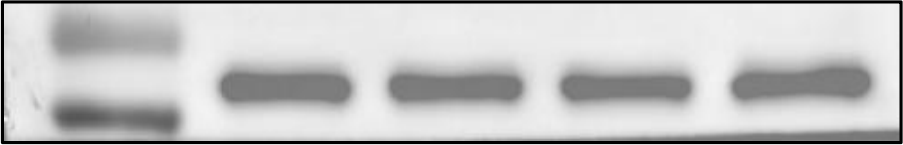


**p-SMAD3**

**58kDa**

**SMAD3**

**58kDa**

**Collagen Ⅰ**

**138kDa**

**α-SMA**

**42kDa**

**GAPDH**

**36kDa**

**Supplementary Figure S1**

Figure 5B


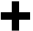
**TGF-β1 CILP (ng/ml)**

**+**

**10**

**+**

**25**

**+**

**50**

**0**

| **p-SMAD3**  **SMAD3**  **Collagen Ⅰ**  **α-SMA**  **GAPDH** | 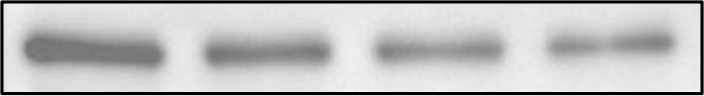  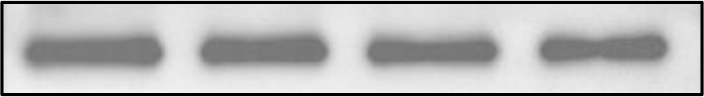  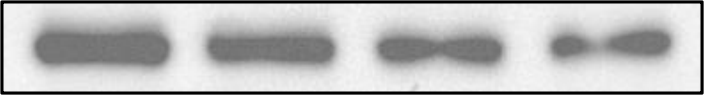  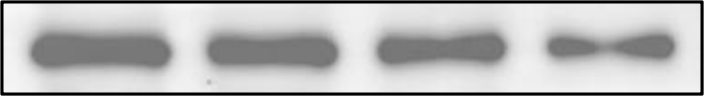  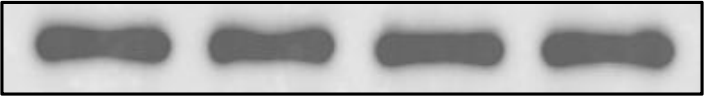 | **58kDa**  **58kDa**  **138kDa**  **42kDa**  **36kDa** |
| --- | --- | --- |

**TGF-β1 CILP (ng/ml)**

**p-SMAD3**

**58kDa**

**SMAD3**

**58kDa**

**Collagen Ⅰ**

**138kDa**

**α-SMA**

**42kDa**

**GAPDH**

**36kDa**


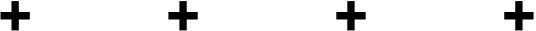


**0 10 25 50**


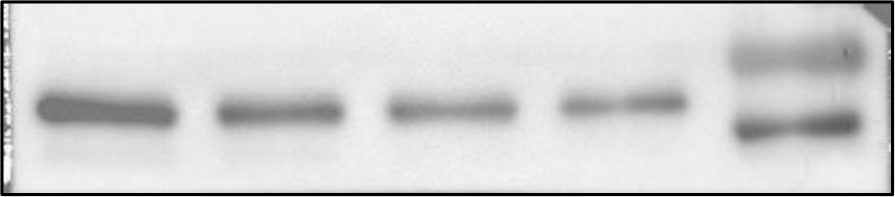


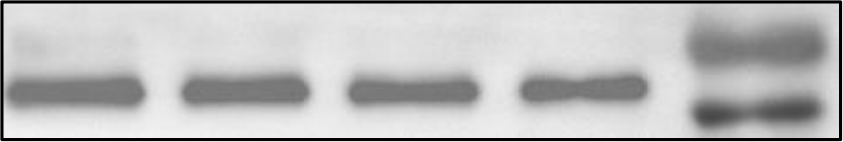


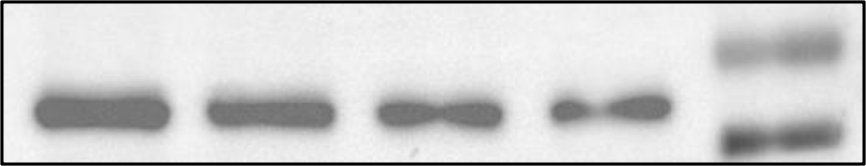


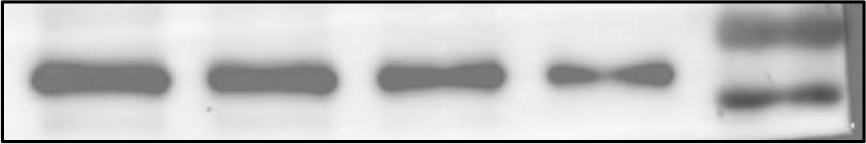


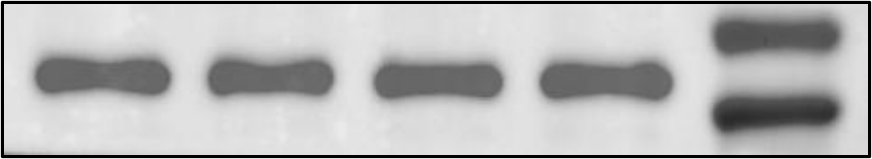


**70kDa**

**55kDa**

**70kDa**

**55kDa**

**150kDa**

**130kDa**

**55kDa**

**35kDa**

**55kDa**

**35kDa**

**Supplementary Figure S1**

Figure 6C

**Non-LFH Group LFH Group**


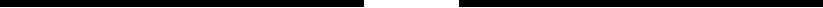


| **SERPINE2**  **GAPDH** | 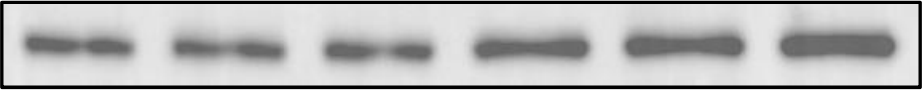  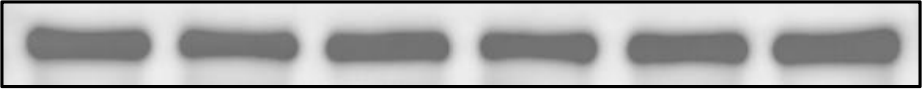 | **45kDa**  **36kDa** |
| --- | --- | --- |

**SERPINE2**

**45kDa**

**GAPDH**

**36kDa**

**Non-LFH Group LFH Group**


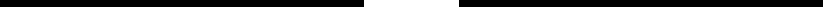


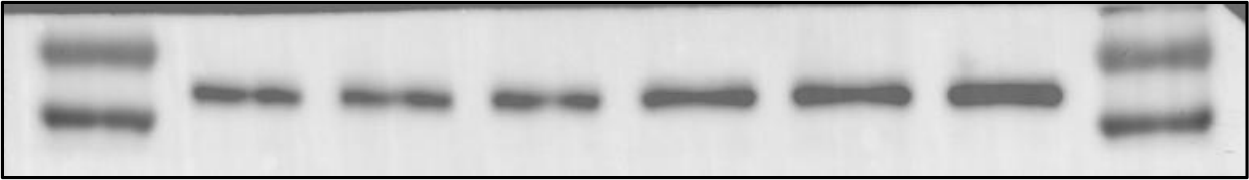


**55kDa 35kDa**


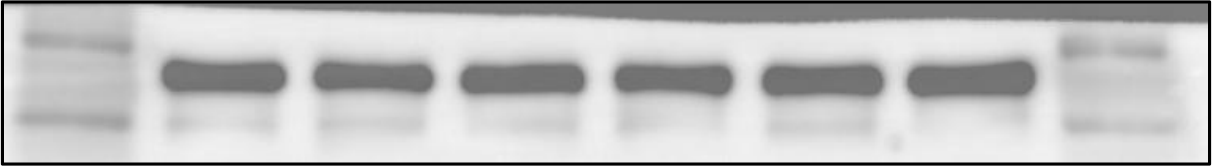


**55kDa 35kDa**

**Supplementary Figure S1**

Figure 6E

**TGF-β1 SERPINE2 GAPDH**

**Con 8h 16h 24h**


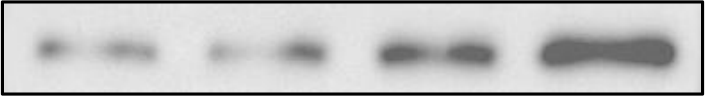


**45kDa**


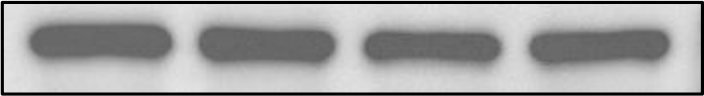


**36kDa**

**TGF-β1 Con 8h 16h 24h**


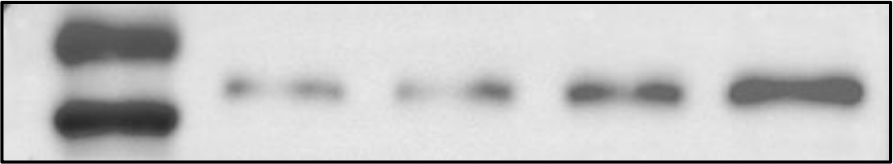


**55kDa 35kDa**


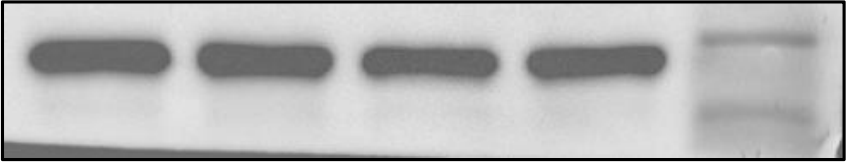


**55kDa 35kDa**

**SERPINE2 45kDa**

**GAPDH 36kDa**

**Supplementary Figure S1**

Figure 6G

**TGF-β1**

**si-SERPINE2 SERPINE2**

**VECTOR-NC**

**CILP**

**p-SMAD3**

**SMAD3**

**Collagen Ⅰ**

**α-SMA**

**GAPDH**

| **-**  **-**  **-**  **+** | **+**  **+**  **-**  **-** | **+**  **-**  **-**  **+** | **+**  **-**  **+**  **-** |
| --- | --- | --- | --- |


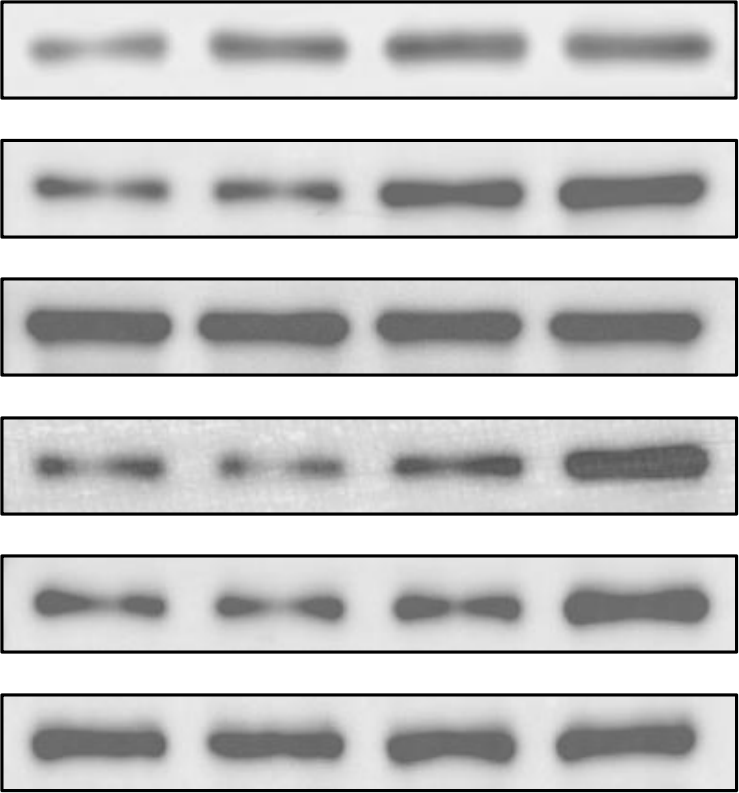


**132kDa**

**58kDa**

**58kDa**

**138kDa**

**42kDa**

**36kDa**

| **TGF-β1 si-SERPINE2 SERPINE2 VECTOR-NC** | **-**  **-**  **-**  **+** | **+**  **+**  **-**  **-** | **+**  **-**  **-**  **+** | **+**  **-**  **+**  **-** |
| --- | --- | --- | --- | --- |


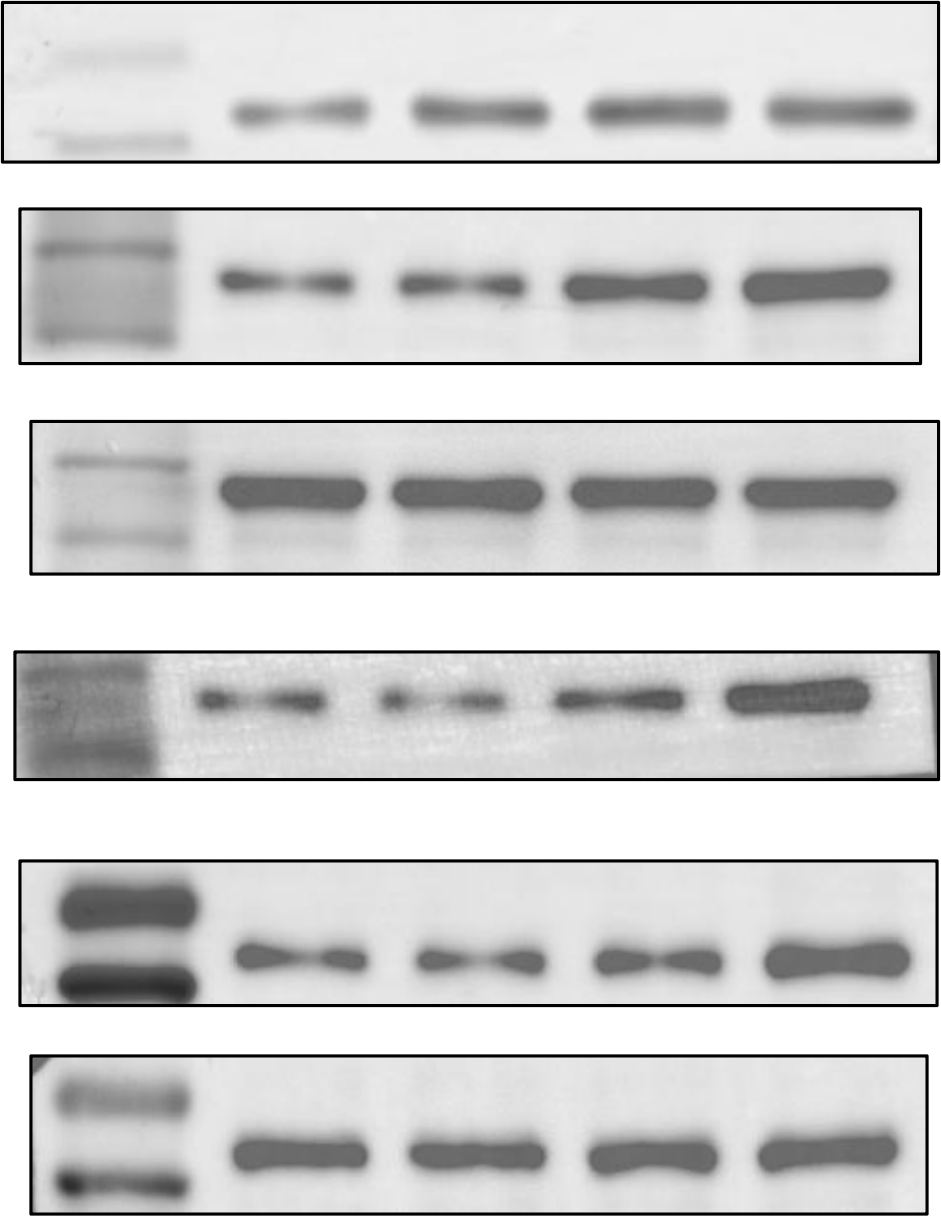


**150kDa**

**130kDa**

**70kDa**

**55kDa**

**70kDa**

**55kDa**

**150kDa**

**130kDa**

**55kDa**

**35kDa**

**55kDa**

**35kDa**

**CILP**

**132kDa**

**p-SMAD3**

**58kDa**

**SMAD3**

**58kDa**

**Collagen Ⅰ**

**138kDa**

**α-SMA**

**42kDa**

**GAPDH**

**36kDa**

**Supplementary Figure S1**

Figure 6I

**TGF-β1 Protein**

**OE-CILP pcDNA3.1 SERPINE2**

**p-SMAD3**

**SMAD3**

**Collagen Ⅰ**

**α-SMA**

**GAPDH**

| **-**  **-**  **-** | **-**  **-**  **+** | **-**  **+**  **-** | **-**  **+**  **+** | **+**  **-**  **-** | **+**  **-**  **+** | **+**  **+**  **-** | **+**  **+**  **+** |
| --- | --- | --- | --- | --- | --- | --- | --- |


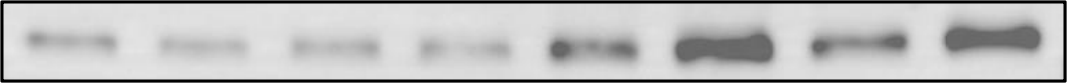


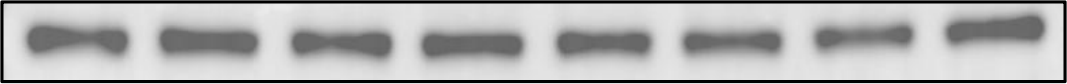


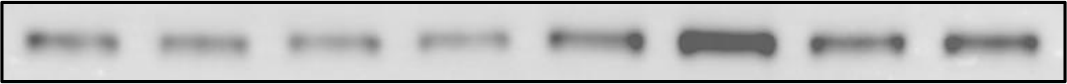


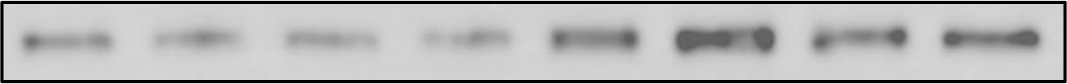


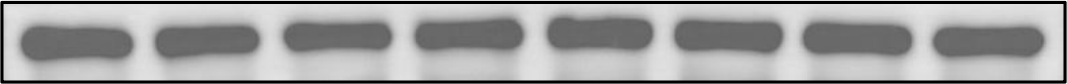


**58kDa**

**58kDa**

**138kDa**

**42kDa**

**36kDa**

| **TGF-β1 Protein OE-CILP pcDNA3.1 SERPINE2** | **-**  **-**  **+** | **-**  **+**  **-** | **-**  **+**  **+** | **+**  **-**  **-** | **+**  **-**  **+** | **+**  **+**  **-** | **+**  **+**  **+** |
| --- | --- | --- | --- | --- | --- | --- | --- |


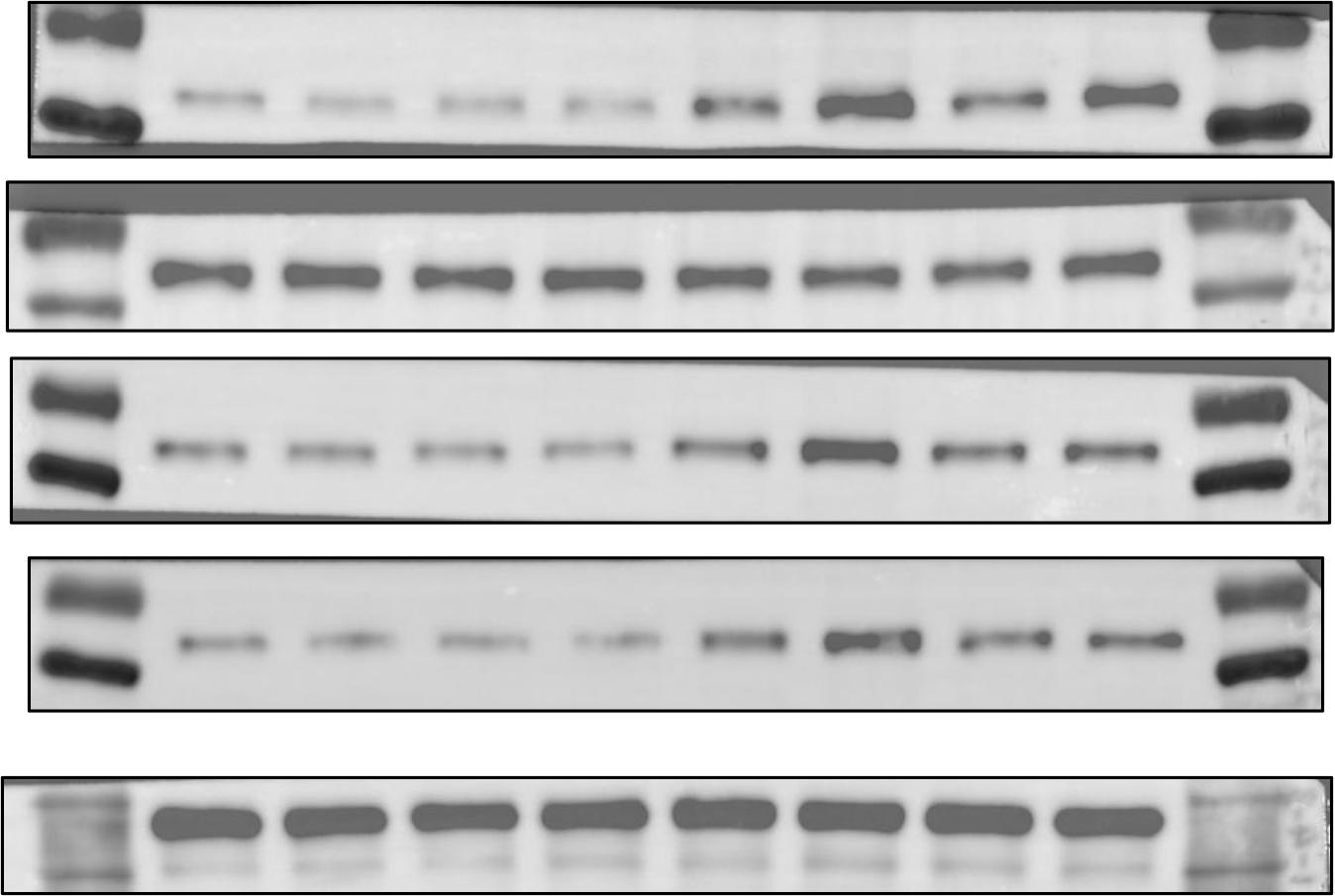


**p-SMAD3 58kDa**

**SMAD3**

**58kDa**

**Collagen Ⅰ**

**138kDa**

**α-SMA**

**42kDa**

**GAPDH**

**36kDa**

**70kDa**

**55kDa**

**70kDa**

**55kDa**

**150kDa**

**130kDa**

**55kDa**

**35kDa**

**55kDa**

**35kDa**

**Supplementary Figure S1**

Figure 6J

**TGF-β1 Protein**

**sh-CILP pcDNA3.1 SERPINE2**

**p-SMAD3**

**SMAD3**

**Collagen Ⅰ**

**α-SMA**

**GAPDH**

**+**

**+**

**+**

**+**

**-**

**-**

**-**

**-**

**-**

**+**

**+**

**+**

**-**

**-**

**-**

**+**

**+**

**+**

**+**

**+**

**-**

**-**

**-**

**-**


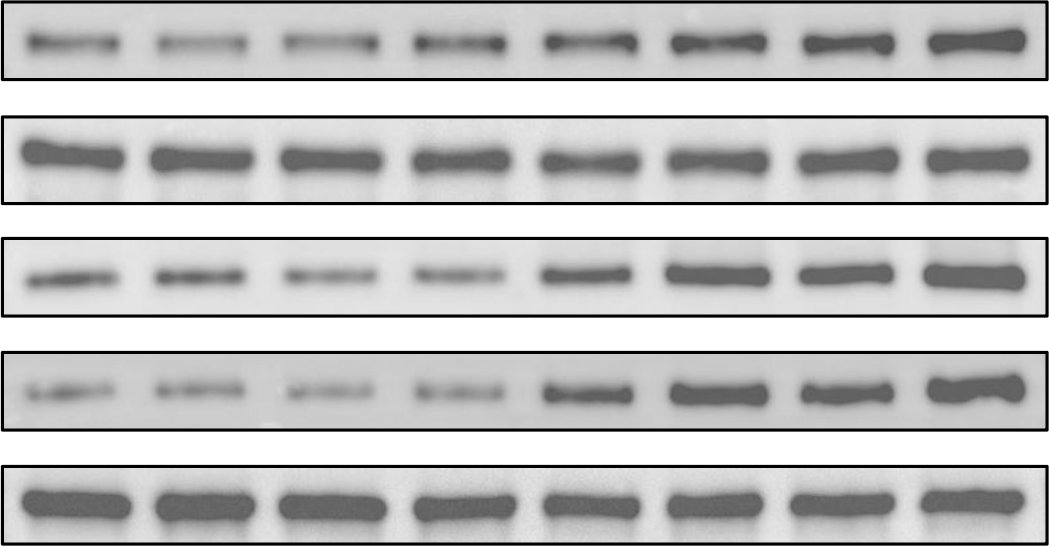


**58kDa**

**58kDa**

**138kDa**

**42kDa**

**36kDa**


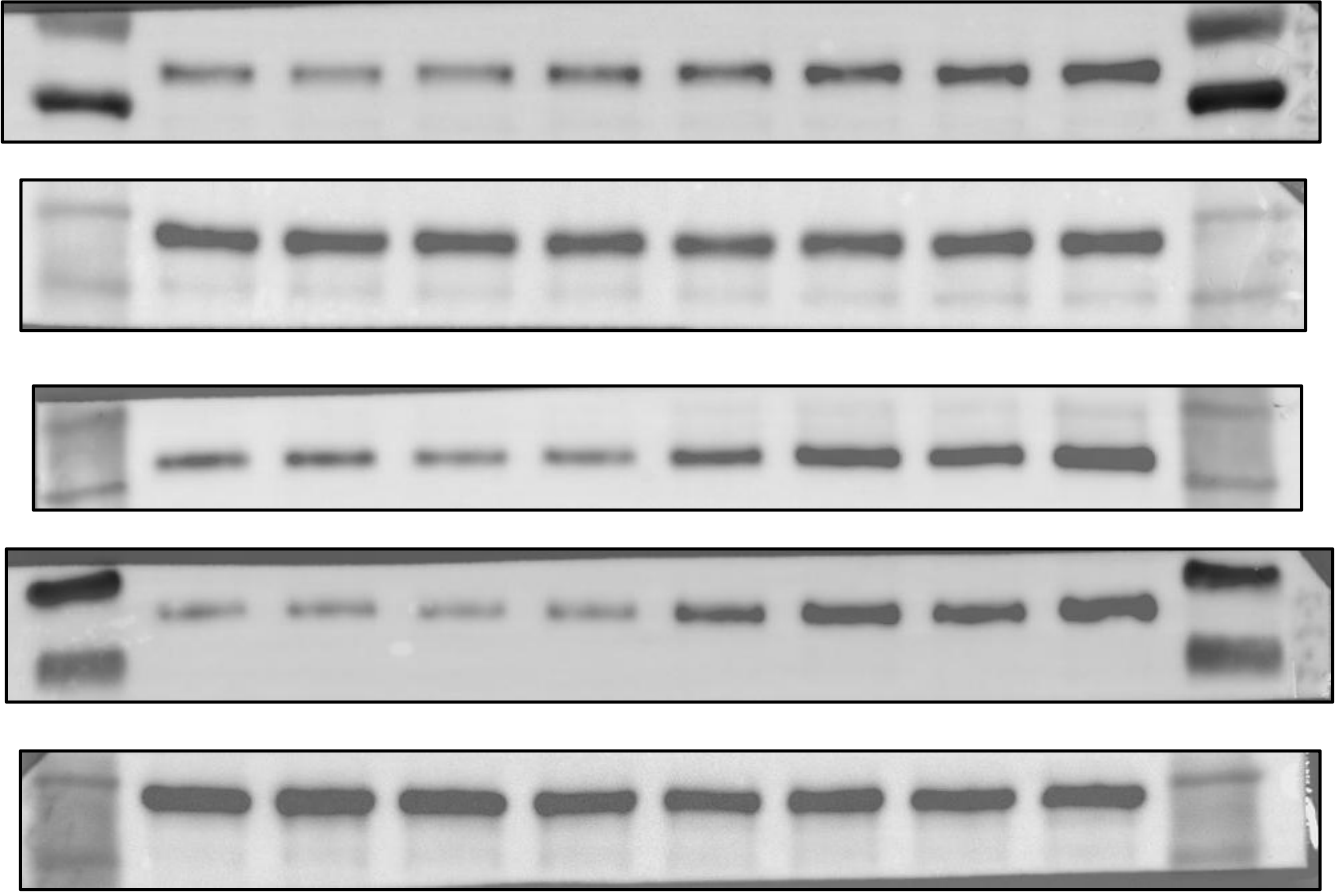
**TGF-β1 Protein sh-CILP pcDNA3.1 SERPINE2**

**+**

**+**

**+**

**+**

**-**

**-**

**-**

**-**

**+**

**+**

**+**

**+**

**-**

**-**

**-**

**-**

**+**

**+**

**+**

**+**

**-**

**-**

**-**

**-**

**p-SMAD3 58kDa**

**SMAD3**

**58kDa**

**Collagen Ⅰ**

**138kDa**

**α-SMA**

**42kDa**

**GAPDH**

**36kDa**

**70kDa**

**55kDa**

**70kDa**

**55kDa**

**150kDa**

**130kDa**

**55kDa**

**35kDa**

**55kDa**

**35kDa**

**Supplementary Figure S2**

CILP


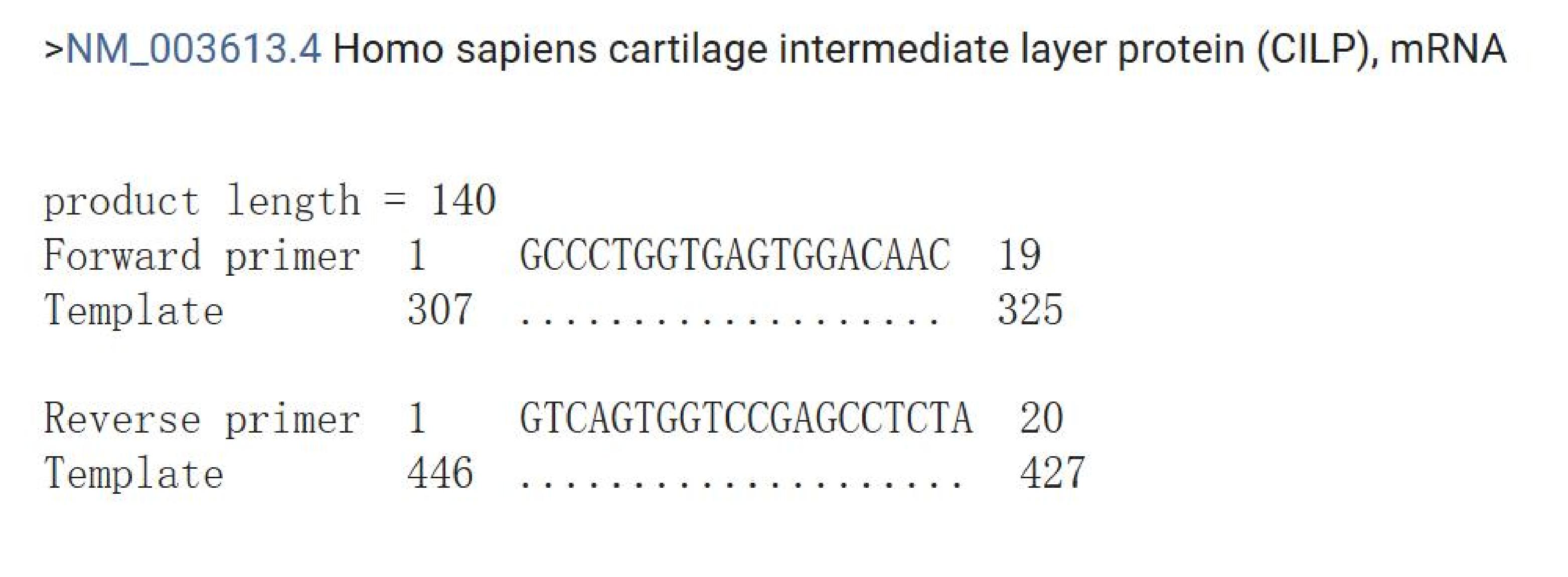


**Supplementary Figure S2**

TGF-β1


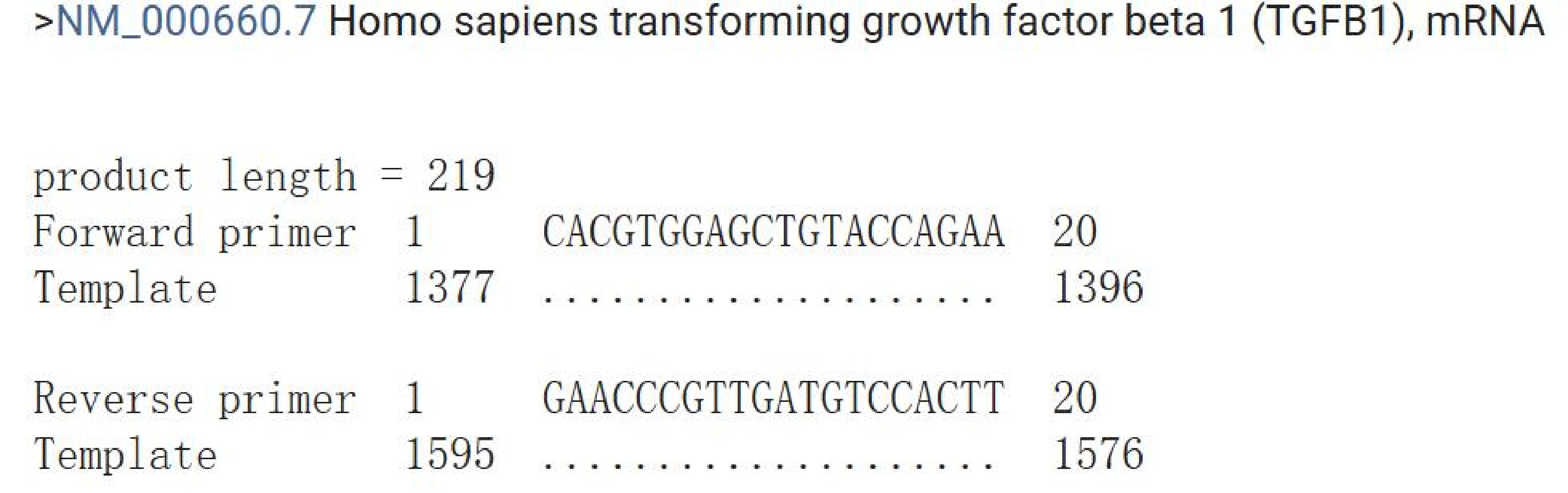


**Supplementary Figure S2**

COL1A2


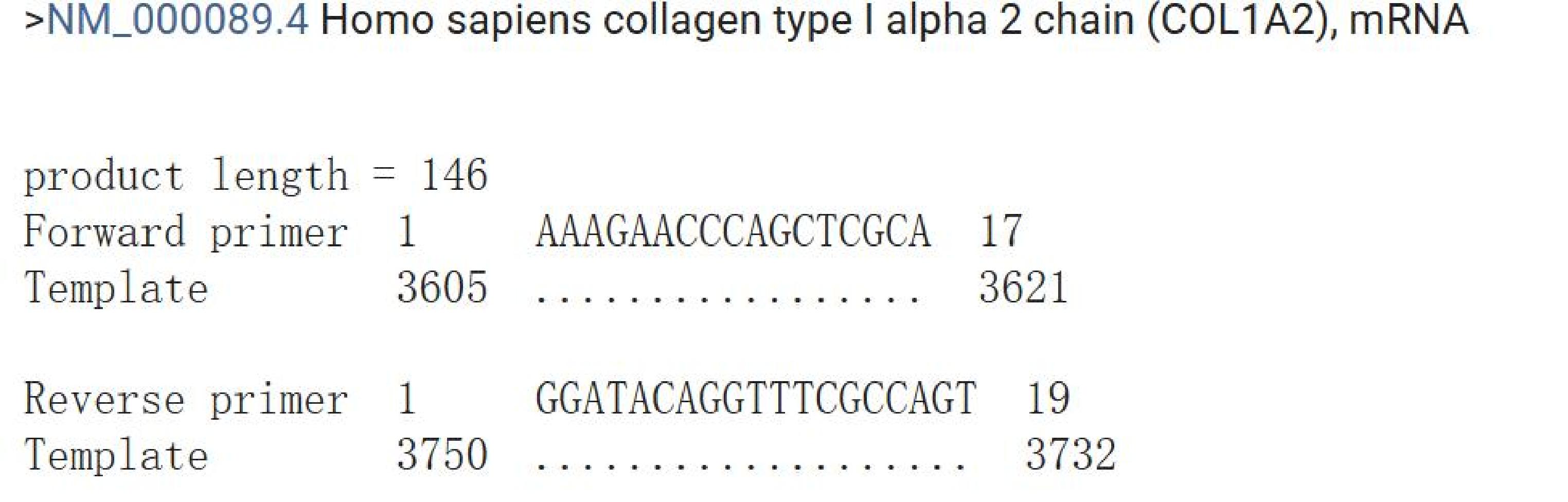


**Supplementary Figure S2**

ACTA2


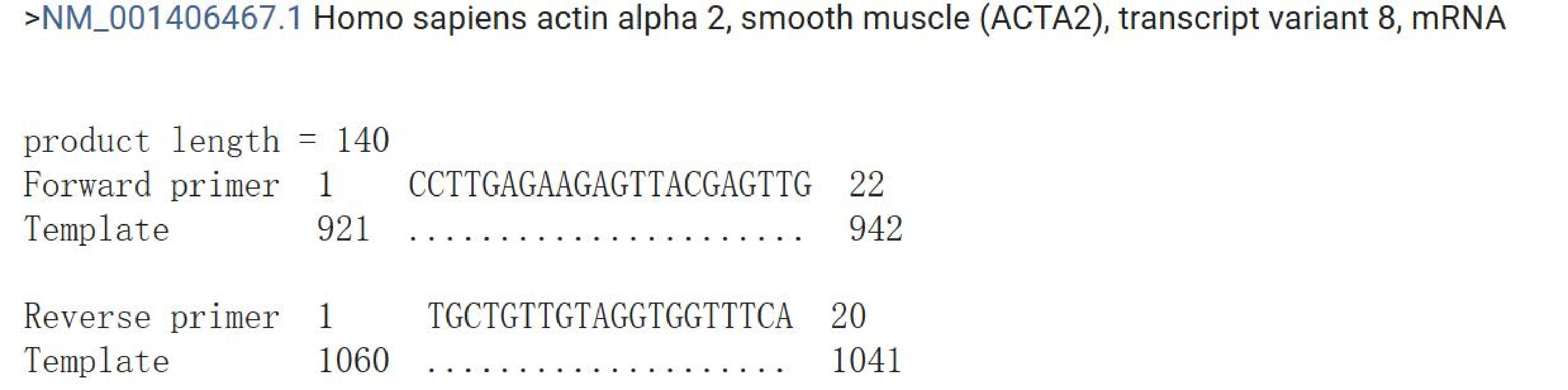


**Supplementary Figure S2**

SERPINE2


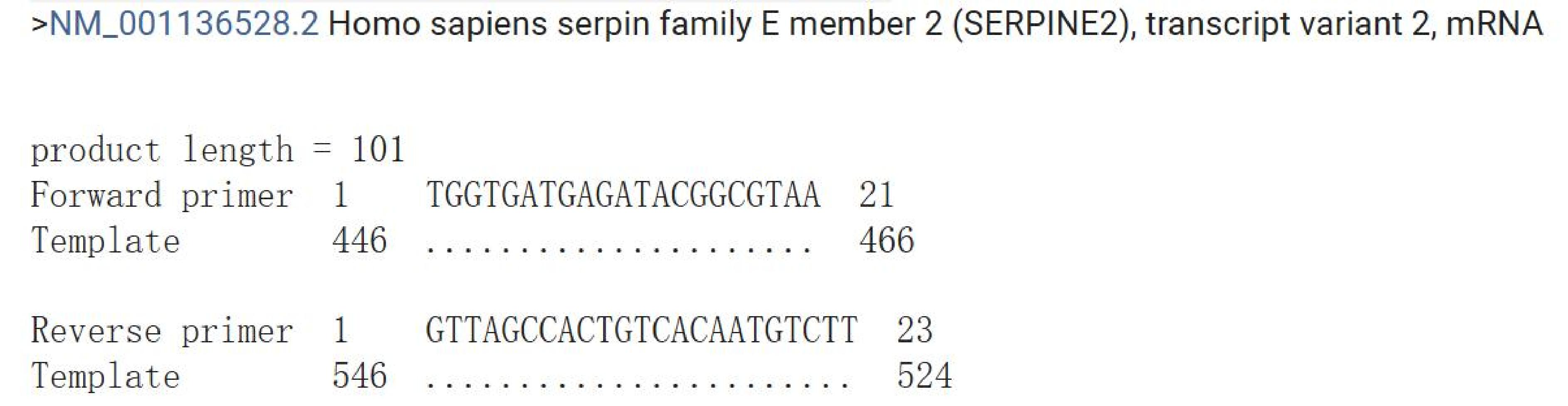


**Supplementary Figure S2**

GAPDH


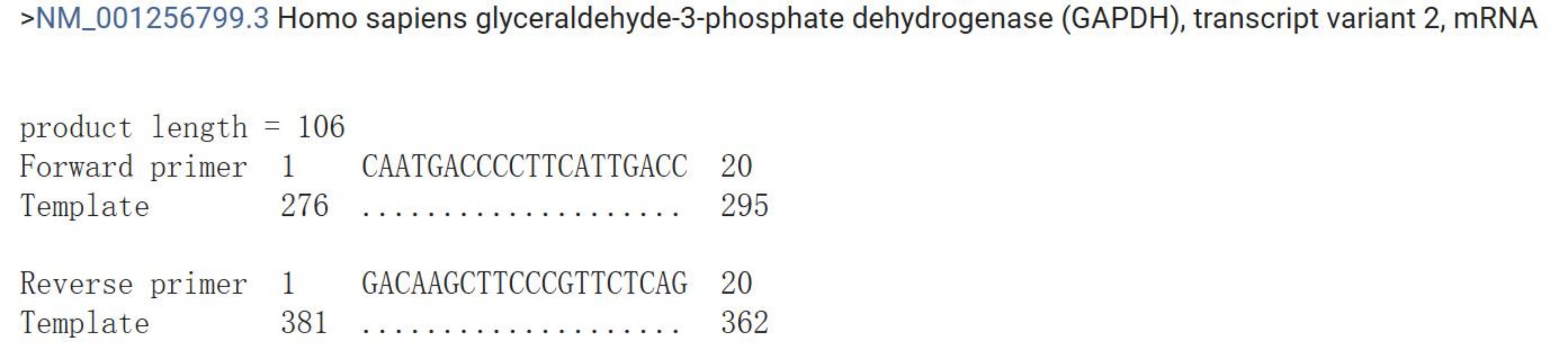

Supplement: Supplementary file 3 — Supplementary Material 3 (DOCX 3.68 MB) [file 18_2025_6051_MOESM3_ESM.docx]
